# Look at the Audience? A Randomized Controlled Study of Shifting Attention From Self-Focus to Nonsocial vs. Social External Stimuli During Virtual Reality Exposure to Public Speaking in Social Anxiety

**DOI:** 10.3389/fpsyt.2021.751272

**Published:** 2021-12-14

**Authors:** Theresa F. Wechsler, Michael Pfaller, Rahel E. van Eickels, Luise H. Schulz, Andreas Mühlberger

**Affiliations:** Department for Psychology, Clinical Psychology and Psychotherapy, University of Regensburg, Regensburg, Germany

**Keywords:** social anxiety, public speaking, attention training, self-focused attention, virtual reality, eye tracking, exposure therapy, cognitive behavioral therapy

## Abstract

**Background:** Enhanced self-focused attention plays a central role in the maintenance and treatment of Social Anxiety and is targeted in contemporary cognitive behavioral therapy. Actual developments use Virtual Reality (VR) for behavioral training. However, no VR attention training combining exposure to public speaking with shifting attention from self-focus to external focus has been investigated, and no experimental evidence exists on different kinds of external cues as targets of attention. Therefore, we investigated the effects of an attention training during public speaking in VR and examined differential effects of an external focus on nonsocial vs. social stimuli.

**Methods:** In this randomized controlled study, highly socially anxious participants were instructed to focus on either objects or the audience within a virtual speech task. We assessed the pre-post effects on affective reactions, self-perception, and attentional processes during public speaking as well as general Social Anxiety using subjective, physiological, and eye-tracking measures. Repeated-measures analyses of variance (ANOVAs) were calculated to detect changes from pretest to posttest over both groups, and time × group interaction effects.

**Results:** Within the analysis sample (*n* = 41), anxiety during public speaking and fear of negative evaluation significantly decreased, with no significant differences between groups. No significant time effect, but a significant time × group effect, was found for the looking time proportion on the audience members' heads. Follow-up tests confirmed a significant increase in the social-focus group and a significant decrease in the nonsocial-focus group. For all other variables, except external focus and fear of public speaking, significant improvements were found over both groups. Further significant time x group effects were found for positive affect during public speaking, with a significant increase in the social focus, and no significant change in the nonsocial-focus group.

**Conclusion:** Our findings suggest that attention training to reduce self-focus can be successfully conducted in VR. Both training versions showed positive short-term effects in the highly socially anxious, with particular advantages of an external social focus concerning eye contact to the audience and positive affect. Further research should investigate whether social focus is even more advantageous long term and if reinterpretations of dysfunctional beliefs could be achieved by not avoiding social cues.

## Introduction

Social Anxiety Disorder (Social Phobia) is characterized by fear or anxiety in, or avoidance of, social situations with the possibility of being scrutinized by others and the fear of acting in a way or showing anxiety symptoms that are negatively evaluated (1). With 12-month prevalence rates ranging between 1.2 and 6.8% (2–7) and lifetime prevalence rates ranging between 2.4 and 7.8% (2, 3, 6), Social Phobia represents a frequent mental disorder. For subthreshold Social Anxiety, 12-month prevalence rates of 3.0% (one *Diagnostic and Statistical Manual of Mental Disorders* [DSM]-IV criterion missing) to 7.5% (two or more criteria missing) were reported in a German mental health survey (7). Within an US population study, the presence of fear of at least one of three public social situations without a substantial interference with the own life or activities was found in 6.7%, interpreted as subthreshold social anxiety (8). Regarding the target of Social Anxiety, the fear of public speaking is very common. A study on social phobia subtypes within a large US population sample found that the speaking fears were the most common of six social fears, and that one third of people with lifetime Social Phobia exclusively reported fear of speaking, while two thirds reported at least one other social fear (9). Within a US student sample, public speaking was selected the most often as a common fear from a list of different fears (10).

Cognitive models of Social Anxiety by Clark and Wells (11), Rapee and Heimberg (12), Hofmann (13), Moscovitch (14), Heimberg et al. (15), and Wong an Rapee (16) proposed different maintenance factors for Social Anxiety, among them anticipatory processing and avoidance behavior before social-evaluative situations, negative social-evaluative cognitions, self-focus, safety behaviors, cognitive avoidance, performance deficits due to anxiety or a lack of social skills, escape behaviors, attentional bias to threat during social-evaluative situations, and post-event processing after social-evaluative situations (16). Wong and Rapee (16) integrated etiological factors for Social Anxiety, like inherited tendencies, parent behaviors, peer experiences, life events, or culture, which proposed to increase the threat value of social-evaluative stimuli. Spence and Rapee (17) also claimed behavior and cognitive aspects like poor social skills, poor social performance, safety behaviors, beliefs, and cognitive processes as proximal factors within a model of genes, temperament, and environmental conditions as developmental factors.

As a specific aspect of biased information processing, the cognitive models of Social Anxiety Disorder describe different deviations in attentional processes as relevant factors in the development and maintenance of Social Anxiety Disorder, which also have been investigated in empirical studies as maintaining factor, causal factor, specifically related factor, or mediator of change in Social Anxiety (18). A prominent attentional bias claimed in all cognitive models (11–16) is enhanced self-focused attention. When entering a social situation, individuals with Social Phobia shift their attention to a detailed monitoring of themselves and their mental representation of how they appear to others. Self-focus leads to an enhanced awareness of own anxiety response, interfering with a realistic processing of the situation and the behaviors of other people. Therefore, the mental representation of the self as seen by the audience is mainly informed by interoceptive information about cognitive, behavioral, or somatic symptoms of anxiety, in which selective external indicators for social evaluation could also be included. In general, self-focus is claimed to increase the threat value of social situations and to maintain Social Anxiety. According to reviews, there is broad empirical support for enhanced self-focused attention in individuals with Social Anxiety Disorder or with high Social Anxiety (16, 18, 19).

Another prominent attentional bias proposed in several cognitive models of Social Anxiety Disorder (12, 15, 16) is selective attention toward social threat cues. Reviews and meta-analyses show empirical support for this bias in Social Anxiety Disorder or high Social Anxiety (16, 18, 20, 21), although potential moderators (18, 20, 21) are discussed, as well as alternative interpretations of results like impaired disengagement from threat cues following initial orientation bias (18, 22). As further bias, an avoidance of social threat cues is also proposed to be associated with Social Anxiety. Within their cognitive model, Clark and Wells (11) mentioned avoidance of social cues as a maintaining factor for Social Anxiety. They suggested that external information on the observers' actual behavior, e.g., gained through eye contact, is avoided, in order to feel in control of the interaction and less vulnerable. Some empirical studies provide support for avoidance of social threat cues, often as a more sustained process following initial hypervigilance (18), resulting in the hypervigilance-avoidance hypothesis (23). Furthermore, there are studies that showed hypervigilant and avoidant attention bias subgroups among socially anxious individuals (24, 25).

It is important to note that the majority of empirical studies examining a hypervigilance or avoidance bias assessed relatively early attentional processes in computerized paradigms using emotional Stroop tasks, modified dot-probe tasks, visual detection tasks, or eye-tracking paradigms with stimulus presentation times of typically 500–1,000 ms, partially shorter (down to 17 ms) or longer (up to 25 s) (18). Only some detection tasks used longer presentation times of up to 5 min (18). In contrast, self-focused attention was mainly examined during social interactions as a whole (18). More naturalistic assessments of avoidance of, or attention toward, social threat cues in Social Anxiety were conducted in recent studies using eye-tracking assessment during public speaking. They showed that socially anxious individuals, compared to controls, avoided looking at the area of the audience (26) or at the faces of the audience members in comparison to nonsocial regions (27). Concerning positive and negative social cues, one study showed that socially anxious individuals compared to controls spent more time looking on faces of socially threatening, in comparison to positive, audience members (28), while another study found that participants with greater fear of public speaking avoided uninterested audience members in comparison to interested audience members (29). Also, Virtual Reality (VR) was already used to examine hypervigilance and avoidance in Social Anxiety. A study by Reichenberger et al. (30) used eye tracking to assess gaze behavior during fear conditioning in VR and found more initial attention toward agents paired with aversive unconditioned stimuli, in comparison to agents paired without, in the highly socially anxious, especially in the first half of fear acquisition, as well as a subsequent avoidance during fear acquisition. In low socially anxious participants, in comparison, no differences were found. To bring them all together, Bögels et al. (18) suggested links between different attentional biases, e.g., that threat stimuli could be internal cues like anxiety symptoms, that hypervigilance toward them might induce self-focused attention, and that avoidance of external social threat cues might mean that attention is more strongly focused on the self.

Coming from cognitive models of Social Anxiety Disorder, cognitive behavioral therapy (CBT) is recommended as a highly efficacious treatment for Social Anxiety Disorder according to the German guideline (31), as well as the British NICE Guideline (32), whereby the latter particularly recommends to offer a CBT referring to the Clark and Wells or Heimberg model. Moreover, exposure therapy, in specific, is claimed as the first-line treatment for anxiety disorders (33, 34), and studies on *in vivo* exposure in Social Phobia show high effect sizes (35). Also, Virtual Reality Exposure Therapy (VRET) can be efficaciously used to treat Social Anxiety. Two meta-analyses found no relevant differences in the efficacy of VRET compared to *in vivo* or imaginal exposure therapy in the treatment of Social Anxiety (36, 37). In contrast, another meta-analysis explicitly comparing VR and *in vivo* exposure in an equal number of exposure sessions, as well as with highly comparable materials and procedures, found a medium effect size indicating superiority of *in vivo* over VR exposure (38). However, they also showed that effect sizes varied a lot over the three included studies on Social Phobia, indicating that VR exposure could be superior but also inferior to *in vivo* exposure. The authors speculate that superiority of VR exposure was reached in one study focusing on reinterpretation while confronting participants with social situations, raising the impression that a combination of VR exposure with cognitive elements might be advantageous. In line with this argumentation, treatment approaches based on the models by Clark and Wells and Heimberg et al. mainly perform exposure for Social Anxiety in the form of experimental tasks, which instruct patients to modulate their attentional focus and/or aim at the correction of dysfunctional beliefs and self-images. Concerning attention focus modulation, in specific, Clark und Wells (11) suggested experimental exercises to demonstrate the adverse effects of self-focused attention and systematic training in externally focused attention as central elements.

Different attentional trainings conducted as stand-alone-treatments or as part of a comprehensive Social Anxiety treatment were already examined concerning their potential to reduce social fears, or respectively to enhance the effects of CBT (18). One prominent attention training approach targets selective attention to threat and promotes an attentional shift away from social threat cues. By guiding patients to direct their attention to nonthreatening aspects of their social environment, those attention bias modification trainings aim at enabling reappraisal of the situation and improvements of proficiency (18). Reviews and meta-analyses provide some evidence for positive effects on Social Anxiety (16, 39–43) but also show inconsistent results and/or potential moderating factors (39–43). The first approaches using VR for attention bias modification (44, 45) found contradictory results as well. Bar-Heim et al. (43) suggested that training attention away from threat could help divert attention from minor threats in the environment, which may be effective in daily circumstances comprising minimal objective threat, but also considered that no clear evidence exists about the effects of attention to multiple elements, in comparison to a single nonthreatening cue. Price et al. (25) discussed a selective effectiveness of this kind of training for socially anxious individuals from a hypervigilant subgroup, but not for individuals from an avoidant subgroup. Further studies hypothesized that an increase in attentional control might explain positive results of those attention bias modification trainings and therefore examined differential effects of attention training away from threat or toward threat. Results were mixed, ranging from increased attentional control and reduced Social Anxiety independently from the trained target of attention (46), over higher reductions of Social Anxiety within the training away from threat (47), to higher reductions within the training toward threat (48). However, since empirical evidence exists for hypervigilance and avoidance biases as relatively fast and automatic processes (18), the relevance of those experimentally examined biases for treatment approaches is still not fully clear.

Another prominent attention training approach for Social Anxiety aims at a reduction of self-focused attention. Within this category of trainings, different ways of shifting the attentional focus away from self-observation are pursued. One kind of training introduced by Wells et al. (49, 50) suggests attention flexibilization exercises in nonthreatening situations to reduce the intensity of self-focus, but also to improve attentional control and attentional breadth. Concerning anxiety reduction in Social Phobia, empirical studies found an advantage of attention flexibilization exercises compared to exercises promoting self-focus (51), but not in comparison to a mindfulness based task (52) and cognitive therapy (53). However, advantages in comparison to mindfulness based and cognitive therapy could be shown concerning a reduction of self-focused attention (52, 53), and in comparison to cognitive therapy also concerning fear of negative evaluation (53). Another kind of training aims at a reduction of self-focus by instructing participants to focus on the task itself during socially threatening situations. A study compared task-focused attention during *in vivo* exposure to plain exposure and found reduced fear of blushing and cognitive changes through both interventions, but significantly higher improvements in cognitive changes at follow-up in the task-focused attention training group (54). Bögels et al. (55) compared task concentration training to applied relaxation, both followed by cognitive therapy, and found a high effectiveness of both treatments with advantages of task concentration training concerning fear of bodily symptoms and dysfunctional beliefs at the interim test before cognitive therapy, and on fear of bodily symptoms at 1-year follow up. The last kind of training aiming at a reduction of self-focus instructs participants to focus on external cues during threatening situations. Woody et al. (56) expanded a CBT group treatment based on the Heimberg model by diaphragmatic breathing exercises and external-focus instructions for social interactions. They found a significant decrease in self-focus attention over time, no change in external focus, and improvement in all outcome variables, with decreased self-focus being associated to reductions in dyad anxiety and self-judgement, but not in speech anxiety, personal fear, general distress, and rater evaluation. Due to a one-group design, the efficacy of attention modulation in specific could not be evaluated. As further examples for studies on the training of an external focus, Feiler et al. (57) showed within a randomized controlled study that an external-focus instruction during a job interview exercise, including observing the interviewers' impressions, led to significant lower interview anxiety and negative self-thoughts in comparison to an internal-focus instruction and a control condition. Positive self-imagery as an alternative intervention strategy showed similar effects concerning interview anxiety, but lower effects concerning negative self-thoughts. Within a single-case series of eight patients, Wells at al. (58) compared plain exposure to simulated or *in vivo* social situations with exposure plus an external-focus instruction including to observe other people. The additional attention instruction showed significant advantages concerning a shift from the observer to the field perspective and from self-focus to external focus and concerning a decrease of anxiety and negative beliefs. Until now, no previous study on attention trainings to reduce self-focused attention in Social Anxiety examined a training realized in VR.

As a relevant point for discussion concerning the training of an external focus to reduce self-focused attention, Wells et al. (58) pointed out the possibility of distraction by focusing on external inanimate stimuli instead of external social stimuli and suggested to examine an exposure-plus-distraction condition in addition to an exposure-plus-external-focus condition. Since focusing on external social cues or external nonsocial cues is both options of shifting one's attentional focus away from the self, socially anxious individuals might prefer to focus on nonsocial stimuli if instructed to direct their attention outwards, as those stimuli may induce less social-evaluative threat. Because focusing away from social stimuli is considered to work as distraction, avoidance, or safety behavior, promoting the maintenance of Social Anxiety, e.g., by inhibiting disconfirmatory processing of dysfunctional beliefs (11, 51, 58), Wells et al. (51, 58) argued that attentional training strategies should be used carefully to not contribute to coping strategies or safety behaviors.

Since no previous study examined the relevance of the kind of external stimuli participants are instructed to focus on when shifting attention away from self-focus, we examined differential effects of attention training for Social Anxiety instructing to focus on external social stimuli vs. external nonsocial stimuli during public speaking as a socially threatening situation. For this purpose, we created the first attention training to reduce self-focused attention realized in VR. To examine the main research question of different kinds of external focus, we instructed participants to direct their attention away from self-focus to either the audience members' faces (external social focus) or objects in the room (external nonsocial focus) during exposure to public speaking in front of a virtual audience. We assessed differences in changes in affective reactions, self-perception, and attentional processes during a speech task conducted at pre- and post-test, as well as in more general measures of Social Anxiety, to test if there are advantages of focusing on social stimuli instead of nonsocial stimuli when shifting the attention away from self-focus to external focus. Since an external focus on nonsocial stimuli might work as avoidance behavior promoting the maintenance of Social Phobia, we expected stronger pre to post improvements in the anxiety level and further affective reactions during public speaking when focusing on social stimuli during the training intervention in comparison to focusing on nonsocial stimuli (Hypothesis 1). Also, for general Social Anxiety, we expected stronger decreases from before to after the social-focus in comparison to the nonsocial-focus training intervention (Hypothesis 2). Furthermore, we expected that a social focus during the training intervention might more strongly improve visual attention toward the audience during talks given after in comparison to before the intervention (Hypothesis 3). Since looking at the audience provides the possibility to verify negative beliefs concerning social evaluation through others and since eye contact represents a quality aspect of the speaking performance and might lead to positive evaluation by others, an increase in visual attention toward the audience also outside the intervention seems to be a desirable outcome of an attention training. For the assessment of visual attention, we fist used VR-based eye tracking to directly measure and examine the participants' proportion of looking time on social and nonsocial stimuli during public speaking before, during, and after the public speaking training. Since no previously published attention training for Social Anxiety was conducted in VR, we furthermore examined the general effectiveness of our VR attention focus training in socially anxious individuals as a secondary research question.

## Materials and Methods

Ethical approval for the study was granted by the ethical review committee of the University of Regensburg (Ref-No.: 17-739-101). The study is reported in accordance with the Consolidated Standards of Reporting Trials (CONSORT) statement (59).

### Study Design

In this randomized controlled study, highly socially anxious participants were randomly assigned to one of two experimental groups receiving different versions of a VR attention training to reduce self-focused attention. According to their group assignment, the participants were trained to direct their attention away from self-focus to external stimuli during a VR exposure to public speaking and in this regard were instructed to focus either on the audience members' faces (group “social focus”) or on objects in the room (group “nonsocial focus”). Before and after the training intervention, we assessed specific aspects of the participants' general Social Anxiety, and they were asked to give diagnostic talks in front of a virtual audience to collect state measures of affective reactions, self-perception, and attentional processes during a speech task. Within a mixed design, we examined changes from pretest to posttest (within-subject effects) and differences in changes between both experimental groups (within-subject/between-subject interaction effects).

### Participants

Nonclinical participants aged between 18 and 35 years with high Social Anxiety indicated by a score of ≥19 on the *Social Phobia Inventory* (*SPIN*) (60) in its German version (61) were included in the study. The SPIN is a self-rating measure that contains 17 questions concerning fear, avoidance, and physiological discomfort in social situations such as attending a party or speaking to an authority. Within a total score ranging from 0 to 68, the authors suggest a score of 19 to be the most appropriate to distinguish between subjects with Social Phobia and those without (60). Exclusion criteria covered mental disorders apart from Social Phobia, Agoraphobia, or Specific Phobias indicated by the *Mini International Psychiatric Interview (M.I.N.I)* (62) in its German version (63). The M.I.N.I. is a structured interview based on the diagnostic criteria of the DSM. We performed the basic version of the M.I.N.I., including affective disorders, anxiety disorders, substance abuse, psychotic episodes, and eating disorders. As further exclusion criterion, individuals with severe physical impairment could not participate in the study.

Participants were recruited through advertisements offering a study on a public speaking training and via a broad screening conducted in classes of first-year undergraduate students at the University of Regensburg. The study took place at the laboratory of the Department of Psychology, Clinical Psychology and Psychotherapy, University of Regensburg.

### Apparatus and Materials

#### VR Environments

**Figure 1:**
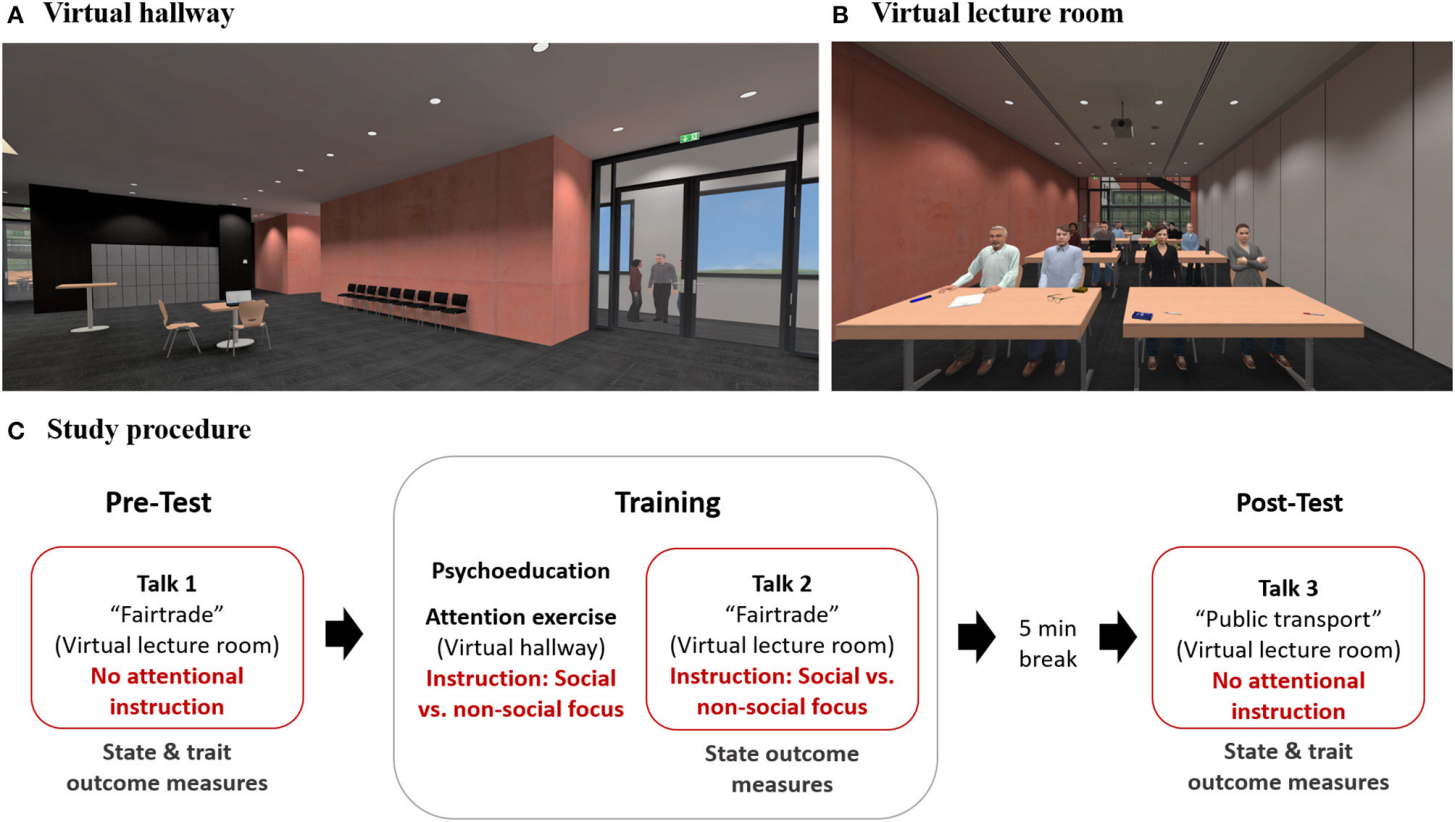
VR environments and study procedure. **(A)** Virtual hallway with inanimate objects and two people in the corridor. **(B)** Virtual lecture room with inanimate objects (tables, windows, walls, items on the tables, etc.) and an audience sitting in rows. Audience members of different sexes and with positive and negative emotional expressions were placed within the first row from left to right: positive/male, negative/male, positive/female, and negative/female. **(C)** Study procedure including a diagnostic speech task at pretest and posttest performed in the virtual lecture room and an attention focus modulation training with psychoeducation, attentional exercises outside public speaking proceeded in the virtual hallway, and an interventional speech task with attentional instruction proceeded in the virtual lecture room. State outcome measures included the assessment of affective reactions (SUD, subjective units of distress; STAI state, State-Trait Anxiety Inventory; PANAS, Brief Measure of Positive and Negative Affect Schedule; BSQ, Body Sensations Questionnaire; HR, Heart rate; SCL, Skin conductance level), self-perception (self-rated effect on others and self-rated appearance), and attentional processes (looking time proportion on heads of the audience, self-focus, and external focus) during public speaking, trait outcome measures included assessments of general Social Anxiety (BFNE, Brief Fear of Negative Evaluation Scale-Revised; PRCS, Personal Report of Confidence as a Speaker).

The virtual environment was generated by the Source SDK (64)-based modification VrSessionMod 0.6 (65). Participants were immersed into VR via a head-mounted display (HMD) of the type HTC Vive (Taoyuan, Taiwan). Experimental control was established using the Software CyberSession 5.8 (66). The virtual environments consisted of two rooms—a hallway and a lecture room. In the virtual hallway, the participants could see objects as well as people in their immediate field of view (Figure 1A). Background noises were audible, consisting of speaking noises and nonverbal noises. In the virtual lecture room, 16 virtual audience members were sitting in altogether four rows, four in each row (Figure 1B). Two female and two male agents were sitting in the first row, with one agent from each sex showing a positive and one showing a negative emotional expression. The agents designed to look friendly and attentive smiled and had an open body position. The agents designed to look pejorative and annoyed had an angry facial expression with narrow eyes, pressed lips, and the corners of the mouth turned down (female and male agents), and folded arms (only female agent). The female and male agents in rows 2 to 4 showed a neutral expression. All virtual audience members were scripted to direct their gaze toward the speaker during the whole talk.

#### Eye Tracking

For eye-tracking measures, an eye tracker (SMI, Teltow, Germany) integrated into the HMD was used. CyberSession 5.8 (66) recorded gaze data at 120 Hz and interpreted it with regard to predefined regions of interest (ROIs) in the virtual environment. ROIs included the audience members' heads, the audience members' bodies below the heads, and the rest of the environment (nonsocial stimuli). For each speech task, the proportion of looking time for valid samples and the dwell time on those ROIs were extracted using MATLAB (67). Results for the categories *head* and *body below head* were additionally summarized into the category *whole body*.

#### Physiological Measurements

Electrodes were placed on the participants' skin for measures of heart rate (HR) and skin conductance level (SCL), amplified via a V-Amp-16 (Brain Products, Gilching, Germany), monitored by the Brain Vision Recorder, and preprocessed with Brain Vision Analyzer 2.1 (both Brain Products, Gilching, Germany). We calculated and extracted mean HR [bpm] and mean SCL [μS] for each segment of interest.

#### Self-Report Measures

A *subjective anxiety scale* (68) based on the rationale of the *subjective units of discomfort scale* (*SUD*) (69) was used to assess the participants' subjective level of anxiety during public speaking ranging from 0 “not at all anxious” to 100 “extremely anxious.”

The state scale of the *State-Trait Anxiety Inventory (STAI)* (70) in its German version (71) was used to assess state anxiety during public speaking. The STAI state contains 20 statements measuring anxiety as a temporary state, resulting in a score ranging from 20 to 80.

The *Brief Measure of Positive and Negative Affect (PANAS)* scale (72) in a German version (73) was used to assess the participants' positive and negative affect during public speaking. The positive affect and negative affect scales each consist of 10 items, and subscores range from 1 to 5.

The *Body Sensations Questionnaire (BSQ)* (74) in its German version (75) was conducted to measure self-reported bodily symptoms during public speaking. The BSQ consists of 17 items, and the total score ranges from 1 to 5.

Questions from a German manual for Cognitive Behavioral Therapy for Social Phobia (76) suggested to accompany experimental exercises in the treatment of Social Phobia were selected and transferred into *self-rating items* to assess *self-attention* and *external attention* during public speaking on an 11-point Likert scale ranging from 0 “not strong at all” to 10 “extremely strong.” For the assessment of external attention, the participants were asked to rate their level of attention focused either on inanimate objects or on people in the audience according to their group assignment. Furthermore, we created items to assess the participants' impression of their own *effect on the listeners* and *appearance in front of the listeners* during public speaking on an 11-point Likert scale ranging from 0 “not good at all” to 10 “extremely good.”

The *Brief Fear of Negative Evaluation Scale—Revised (BFNE-R)* (77) in its German version (*Furcht vor Negativer Evaluation—Kurzskala* (*FNE-K*)) (78) was used to measure fear of negative social evaluation, consisting of 12 questions regarding the fear of being negatively evaluated in various social occasions. The total score ranges from 12 to 60 (79).

The 30-item version of the *Personal Report of Confidence as a Speaker (PRCS)* (80) in a German version (81) was used to measure fear of public speaking as a further aspect of general Social Anxiety. The 30 items are answered in a true-or-false format, resulting in a total sum score ranging from 0 (all responses *false*) to 30 (all responses *true*).

The *Igroup Presence Questionnaire (IPQ)* (82) in its German version *Fragebogen zum Erleben in Computerwelten* (83) was used to measure the participants' sense of presence experienced in the virtual environment.

*Rating items* were used to assess the perceived valence of the emotional expressions of the female and male audience members sitting in the first row (Figure 1B). The participants were asked to rate the intensity of the emotional expressions *friendly, attentive, pejorative*, and *annoyed* on an 11-point Likert scale ranging from 0 “not at all” to 10 “extremely.” Mean values for positive and negative emotional expressions were calculated by averaging the values for friendly and attentive for positive valence and pejorative and annoyed for negative valence.

#### Speech Task

To assess the participants' affective reactions, self-perception, and attentional processes during public speaking, we conducted two diagnostic speech tasks, one before (pretest) and another after (posttest) the training intervention (Figure 1C). The public speaking tasks consisted of 5-min talks on the topic “Fairtrade food” (pretest) and “public transportation” (posttest) and were given in front of a virtual audience in a virtual lecture hall (Figure 1B). Beforehand, the participants received an information sheet on pro and con arguments concerning the respective topic and had a short preparation time. They did not receive a specific instruction concerning their attentional focus for the diagnostic talks.

### Intervention

The one-session VR attention training combines a VR exposure to public speaking with training to shift attention from self-focus to external focus. It includes psychoeducation, attention exercises outside public speaking, and attention modulation during an exposure to public speaking. One version of the training instructs participants to focus externally on people (social focus), and the other version on objects in the room (nonsocial focus). The trainings were conducted by two female psychology students, trained to apply the intervention to highly socially anxious participants and supervised by a licensed psychotherapist.

For psychoeducation, participants received written information about the development, maintenance, and possibilities for the modification of Social Anxiety according to the Cognitive Model of Social Phobia by Clark and Wells (11). They were informed that reducing self-focused attention and instead focusing externally during public speaking may help to reduce Social Anxiety.

During the attention focus exercise, the participants practiced the modulation of their attention focus outside public speaking. The exercise took place in a virtual hallway (Figure 1A), where the participants first received a voice-over instruction to observe and describe noises they could hear, either people talking (social-focus group) or other noises (nonsocial-focus group). Secondly, they were instructed to observe and describe either people (social focus group) or objects (nonsocial-focus group) they could see in the hallway. The exercise is based on the suggestions for the treatment of Social Phobia by Clark and Wells (11) and follows an exercise suggested by a related German treatment manual (76). Adaptions for the here-conducted training include a transfer into VR and a splitting into an attention modulation with a focus on social stimuli and nonsocial stimuli.

As the main part of the attention focus training, we instructed the participants to shift their attention from self-focus to external stimuli during public speaking. This part of the training took place in the virtual lecture hall (Figure 1B), where the participants had to give a 5-min talk on “Fairtrade food” in front of a virtual audience. They already gave a talk on this topic during the diagnostic speech task at pretest and now were asked to repeat their talk and, while doing so, to shift their attention away from self-focus to external stimuli. According to their group assignment, the participants were instructed to either focus on objects they could see in the virtual lecture hall, e.g., tables, windows, or walls (nonsocial-focus group), or on the people in the audience, specifically their faces and facial expressions (social-focus group).

### Outcomes

To examine both research questions and to test the hypothesis for the main research question (see Introduction), we examined the effects of the two VR attention focus training versions on three outcome areas: state measures of affective reactions and self-perception during public speaking, state measures of attentional processes during public speaking, and measures of general Social Anxiety.

As a primary outcome concerning state measures of affective reactions and self-perception during public speaking, we assessed the participants' mean subjective level of anxiety during public speaking using SUDs. SUDs were chosen as the primary outcome for the research questions and for Hypothesis 1 since they could be assessed directly during the talks in VR. We collected this measures verbally at the beginning, after the first minute, after the fourth minute, and after finishing the 5-min speech tasks performed for diagnostic reasons at pretest and posttest and for interventional reasons during the attention modulation training. The mean SUD value was computed over all four single values for each talk. As secondary outcomes regarding affective reactions, we assessed state anxiety using the STAI state, positive and negative affect using the PANAS, body sensations using the BSQ, and HR and SCL. As additional secondary outcomes as regards the participants' self-perception during public speaking, the self-rated effect of own person on others and the self-rated appearance of own person in front of others were assessed. All outcomes were measured during or directly after the three speech tasks (Figure 1C).

As a primary outcome concerning attentional processes during public speaking, the participants' attention to the audience members' faces was measured via eye tracking during all three talks, operationalized by the proportion of time spent looking on the audience members' heads. This variable was chosen as primary outcome for the research questions and Hypothesis 3 because an observation of the audience members represents an exposure to social stimuli, probably promoting cognitive reinterpretations of dysfunctional beliefs (11). Furthermore, visual contact to the audience can be considered as a quality aspect of the speaking performance. As secondary outcomes regarding attentional processes, we assessed self-focused attention and externally focused attention during public speaking via self-rating items collected directly after the three speech tasks (Figure 1C).

As a primary outcome concerning general Social Anxiety, we analyzed the fear of negative evaluation measured via the BFNE. This variable was chosen as primary outcome for the research questions and Hypothesis 2 as a previous study showed advantages of attentional training in comparison to cognitive therapy for fear of negative evaluation (53), and therefore the effects of two different attentional training versions on this variable are of interest. As a secondary outcome, the participants' fear of public speaking was measured via the PRCS. We measured both variables before and after the training intervention (Figure 1C).

For a manipulation check, we also assessed the participants' attention toward nonsocial stimuli during public speaking. For ancillary analysis, we assessed the participants' attentions toward the heads of the audience members in the first row (Figure 1B), representing individuals from different sexes and with different emotional expressions. To capture attention toward social stimuli comprehensively, we furthermore measured attention toward the audience members' whole bodies in addition to attention toward their heads only. All those attention-related variables were measured during all three speech tasks via eye tracking and operationalized as a proportion of looking time on the respective ROIs.

As covariates, we measured the perceived valence of the emotional expressions of the audience members sitting in the first row via rating items and the participants' presence in VR using the IPQ, both assessed at the end of the experiment.

### Procedure

Fist, the SPIN was conducted as a screening measure for Social Anxiety, and only participants with a score ≥19, and fulfilling further general inclusion criteria, were invited to participate in the study. After receiving detailed information on the study procedure, eligible participants gave written consent to their participation. After a questionnaire on sociodemographic and health characteristics, the M.I.N.I. interview was conducted. Only participants not fulfilling the criteria for a mental disorder diagnosis defined as the exclusion criterion were randomly assigned to one of the two training versions and passed on for the further study procedure (Figure 1C). This included the pretest assessments of BFNE and PRCS. Afterwards, electrodes for psychophysiological measurements were placed. The participants then entered the VR to get comfortable with the experience. After taking off the HMD, participants received instruction on the pretest speech task and had a short preparation time. After entering the VR again, participants gave their 5-min diagnostic talk on the topic “Fairtrade”, while eye tracking and physiological measures were conducted and a voice-over was asking for SUD ratings at several time points. There was no instruction given concerning the attentional focus during public speaking. After leaving the VR, questionnaires measuring state outcomes related to the talk were assessed. Afterwards, the participants received the training intervention according to their group assignment, including psychoeducation, the attention exercise in the virtual hallway, and the attention modulation during exposure to public speaking in the virtual lecture room. During the interventional speech task, participants repeated their talk on “Fairtrade,” with the explicit instruction to either focus on social stimuli or nonsocial stimuli according to their group assignment. Eye tracking and physiological measures, as well as SUDs, were again collected during the talk. After leaving the VR, participants completed questionnaires on the state measures once more and were then asked to leave the room to take a break of 5-min duration. After they returned, the posttest measurements were conducted, starting with the assessment of the BFNE and PRCS. Then, the posttest speech task was conducted. As in the first diagnostic talk, without an explicit instruction concerning their attentional focus, they had some preparation time, then entered the virtual lecture room, and gave a 5-min talk on “public transport”. The measurements of outcome variables at posttest were equivalent to those of the pretest. Finally, the IPQ and a rating of the valence of the audience members' emotional expressions were conducted.

### Randomization

The participants were assigned randomly and in equal number to two active experimental groups by use of a computer-generated randomization list.

### Blinding

All participants received the information that an attentional shift away from self-focus to external stimuli may help to reduce Social Anxiety during public speaking. They were not aware of the respective other instruction concerning the concrete content of external focus (social vs. nonsocial focus) and were not informed of the hypotheses of a potential advantage of the social focus. The experimenter was aware of the group assignment from the beginning of the experiment.

### Missing Values

If no more than two items within one questionnaire were omitted, missing values were replaced with the arithmetic mean of the available data within the respective questionnaire (84). One missing item occurred in one case for STAI post, BSQ post, PANAS negative affect intervention, PANAS positive affect pre and intervention, each; in two cases for BSQ pre, PCRS pre, PCRS post, and IPQ; and in four cases for BSQ intervention. More than two missing items did not occur in any questionnaire; therefore, we did not have to exclude participants from the analysis of questionnaire-based outcome measures. For the physiological measures HR and SCL, missing data occurred in two participants due to technical problems. The respective participants, one from each experimental group, were excluded from the analysis of the physiological outcome variables. For the eye-tracking measures, there were missing data for the posttest in one participant from the nonsocial-focus group, being excluded from the analysis of eye-tracking outcomes.

### Statistical Methods

First, descriptive statistics were calculated for all sociodemographic, health, and VR presence variables. Second, group differences between those sample characteristics were calculated using *t*-tests, chi-square tests, or Fisher's exact test. Third, group differences at pretest were checked for all outcome variables using *t*-tests. Fourth, we conducted 2 × 2 repeated-measures analyses of variance (ANOVAs) for all primary, secondary, and ancillary outcome variables with time (pretest; posttest) as the within-subject factor and group (social focus; nonsocial focus) as the between-subject factor and examined the results for significant time effects and significant time × group interaction effects. As additional analyses, we conducted independent-sample *t*-tests to check for significant group differences at posttest and, in the case of significant time × group effects, additional dependent-sample *t*-tests to check for significant changes from pretest to posttest separately in both groups. Fifth, we conducted a manipulation check by calculating 2 × 2 repeated-measures ANOVAs with time (pretest; intervention) as the within-subject factor and group (social focus; nonsocial focus) as the between-subject factor for the attention-related outcome variables and examined the results for significant time effects and significant time × group effects. Again, additional independent-sample *t*-tests for group differences at the intervention were conducted and, in the case of significant time × group effects, additional dependent-sample *t*-tests for changes between pretest and intervention separately for both groups. Sixth, as auxiliary analyses, we conducted descriptive statistics on the valence ratings for the emotional expressions of the audience members sitting in the first row over all participants. Seventh, we analyzed changes in affective reactions and self-perception during public speaking from the pretest to the interventional speech task, to examine differences in the reactions toward the two versions of the intervention. Therefore, we conducted 2 × 2 repeated-measures ANOVAs with time (pretest; intervention) as the within-subject factor and group (social focus; nonsocial focus) as the between-subject factor and examined the results for significant time effects and time × group effects. Eighth, we analyzed changes and differences in changes between groups from intervention to posttest to examine the maintenance of changes in affective reactions, self-perception, and attentional processes beyond the intervention. Therefore, we conducted 2 × 2 repeated-measures ANOVAs for all outcome variables with time (intervention; posttest) as the within-subject factor and group (social focus; nonsocial focus) as the between-subject factor and examined the results for significant time effects and significant time × group effects. Complementing the analyses of proportions of looking time (see also Limitations), all analyses for eye-tracking-related outcomes were additionally conducted for the dwell time as another outcome variable and are reported in Supplementary Tables 1, 2.

We considered *p*-values ≤0.050 as significant for two-tailed analyses. For the main analyses, *p*-values ≤0.100 were reported as trendwise effects. We calculated $\eta_{\text{p}}^{2}$ as effect size measures, interpreted as small effect (0.01), medium effect (0.06), and large effect (0.14) referring to Cohen (85). If the Levine test indicated inhomogeneity of variances, corrected values for independent-sample *t*-test following the Welch correction are reported. If nonsphericity was indicated, corrected values for ANOVA results following the Greenhouse–Geisser correction are reported. Data analyses were performed using SPSS 26 (86), R (87), knitr (88), tidyverse package v1.3 (89), and MATLAB (67).

## Results

### Participant Flow

From 49 participants assessed for eligibility, one was excluded due to a mental disorder diagnosis specified as the exclusion criterion. Therefore, 48 participants were randomly assigned to either the nonsocial-focus condition (*n* = 24) or the social-focus condition (*n* = 24). Due to technical problems, three participants from each experimental group could not receive the full intervention. The VR presentation broke down while those participants were giving their talk and could not be restarted at the same day; thus, their examination could not be finished. One participant from the nonsocial-focus condition had to be excluded for analyses due to lost questionnaires.

### Sample Characteristics

Table 1 displays sociodemographic, health, and VR presence characteristics for the analysis sample consisting of 41 participants, separated into participants from the nonsocial-focus condition (*n* = 20) and the social-focus condition (*n* = 21). Group comparisons did not show significant differences in the sample characteristics between both experimental groups (Table 1). Furthermore, no significant group differences were found in the outcome variables at pretest, except of higher mean values for state anxiety (STAI state) and fear of public speaking (PRCS), and a lower mean value for the self-rated appearance in the social-focus group (Supplementary Table 3).

**Table 1:** Sociodemographic, health, and VR-related sample characteristics.

**Characteristics**	**Nonsocial-focus condition (*n* = 20)**	**Social-focus condition (*n* = 21)**	***t*/χ^2^/FE**	***p***
**Sociodemographic variables**				
Female sex, *n* (%)	16 (80.0)	20 (95.2)	2.22	0.184
Age, *M* (*SD*), years	20.10 (1.52)	21.09 (3.25)	−1.26	0.216^d^
General qualification for university entrance, *n* (%)	20 (100)	21 (100)	n.a.	n.a.
Highest professional qualification, *n* (%)			FE	0.184
Still a student or in vocational training	19 (95.0)	16 (76.2)		
Other^a^	1 (5.0)	5 (23.8)		
Financial situation, *n* (%), €/month			FE	0.606
≤1,000	18 (90.0)	20 (95.2)		
>1,000	2 (10.0)	1 (4.8)		
Not in a relationship, *n* (%)	14 (70.0)	10 (47.6)	2.11	0.146
Living alone, *n* (%)^b^	5 (27.8)	5 (25.0)	0.04	0.846
Living area, *n* (%)^c^				
Rural/suburban	8 (42.1)	8 (38.1)	FE	0.796
Urban	11 (57.9)	13 (61.9)		
**Mental health variables**				
Social Anxiety (SPIN total), *M* (*SD*)	24.15 (5.31)	27.76 (7.40)	−1.79	0.082
Current diagnosis of Social Phobia, *n* (%)	2 (10)	3 (14.3)	FE	1.000
Current diagnosis of agoraphobia, *n* (%)	1 (5.0)	0 (0.0)	FE	0.488
Past mental disorder diagnosis, *n* (%)	1 (5.0)	3 (14.3)	FE	0.606
Past psychotherapeutic treatment, *n* (%)	1 (5.0)	3 (14.3)	FE	0.606
Past psychopharmacological treatment, *n* (%)	1 (5.0)	1 (4.3)	FE	1.000
**VR-related variables**				
Spatial Presence (IPQ subscale), *M* (*SD*)	3.75 (0.81)	4.20 (0.74)	−1.87	0.069
Involvement (IPQ subscale), *M* (*SD*)	3.57 (1.46)	3.63 (1.33)	−0.13	0.898
Experienced Realism (IPQ subscale), *M* (*SD*)	3.13 (0.93)	2.86 (0.78)	1.00	0.324
General Presence (IPQ subscale), *M* (*SD*)	4.20 (1.15)	4.30 (1.03)	−0.29	0.774

*The table displays mean values (M) and standard deviations (SD) for metric variables and absolute (n) and relative (%) frequencies for categorical variables, t-values and p-values from t-tests conducted to examine group differences in metric variables, and χ^2^-values and p-values from chi-square-test, or—if too few cases in certain categories—p-values from Fisher's exact tests (FE), both conducted to examine group differences in categorical variables. SPIN, Social Phobia Inventory; IPQ, Igroup Presence Questionnaire; n.a. not applicable*.

a *Including completed vocational training/master/technician/comparable, university degree/university of applied sciences degree, or no professional qualification*.

b *Three missing values, n = 38*.

c *One missing value, n = 40*.

d *Welch correction due to inhomogeneity of variances according to the Levine test*.

### Main Results

**Figure 2:**
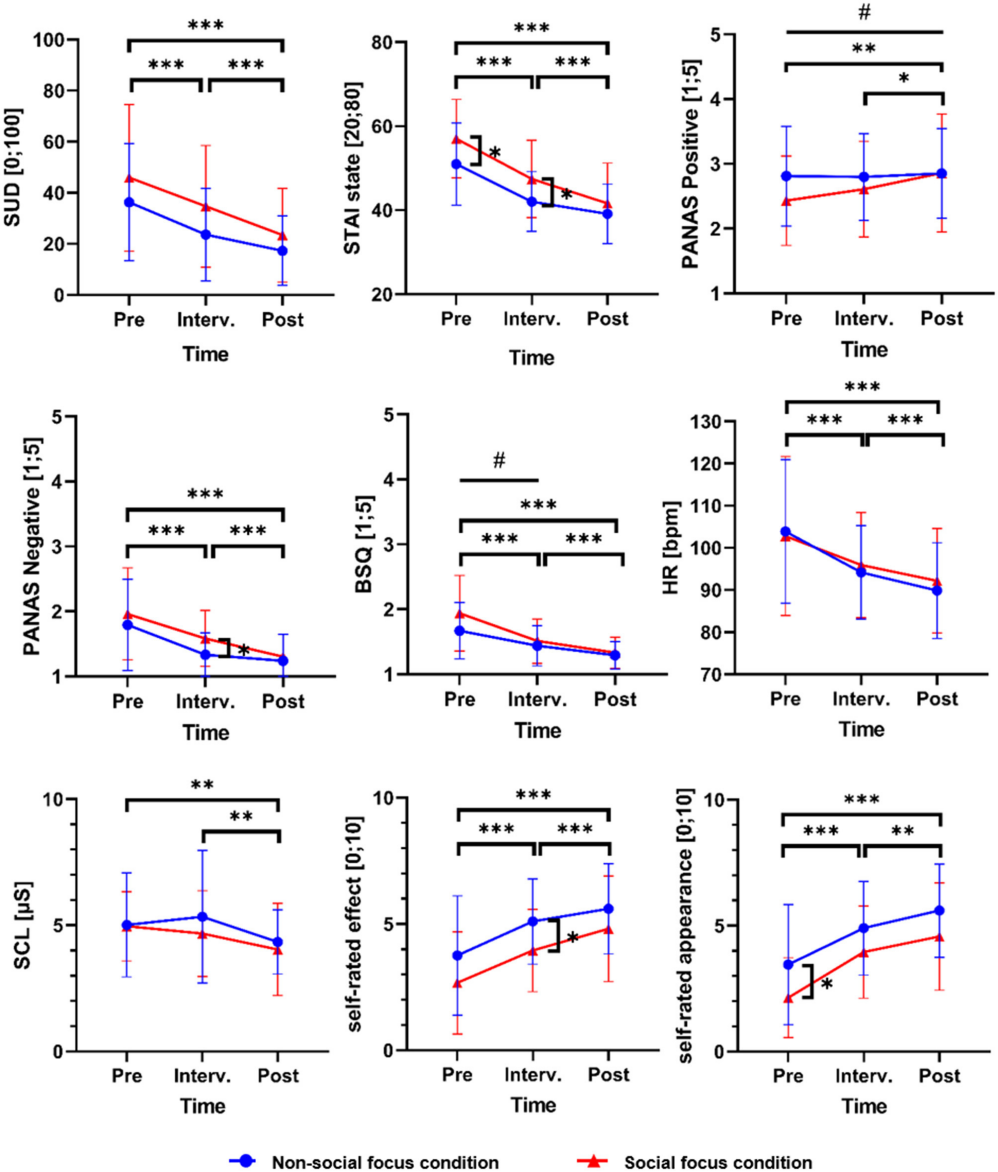
Changes in affective reactions and self-perception during public speaking over all speech tasks separately for both training groups. The figure displays means (indicated by circle and triangle symbols) and standard deviations (indicated by error bars) for affective and self-perception-related state outcome variables during the speech tasks at pretest, intervention, and posttest, separately for the nonsocial-focus group (*n* = 20) and the social-focus group (*n* = 21). Vertical square brackets with asterisks indicate significant group differences at pretest, intervention, or posttest (as indicated by independent-sample *t*-tests; Supplementary Table 3). Horizontal square brackets with asterisks indicate significant main effects of time between pretest and the intervention, the intervention and posttest, or pretest and posttest, and lines with hashtags indicate significant time × group interaction effects between the respective time points (as indicated by repeated-measures ANOVAs; Table 2 and Supplementary Table 2). */#*p* ≤ 0.050; **/##*p* ≤ 0.010; ***/###*p* ≤ 0.001. SUD, subjective units of distress; STAI state, State-Trait Anxiety Inventory; PANAS, Brief Measure of Positive and Negative Affect Schedule; BSQ, Body Sensations Questionnaire; HR, heart rate; SCL, skin conductance level. One missing value for HR and SCL in the nonsocial-focus group, *n* = 19, and in the social-focus group, *n* = 20. Numeric data on mean values and standard deviations are displayed in Supplementary Table 1. The results on changes between pretest and intervention are additionally described in Supplementary Text 2.

**Figure 3:**
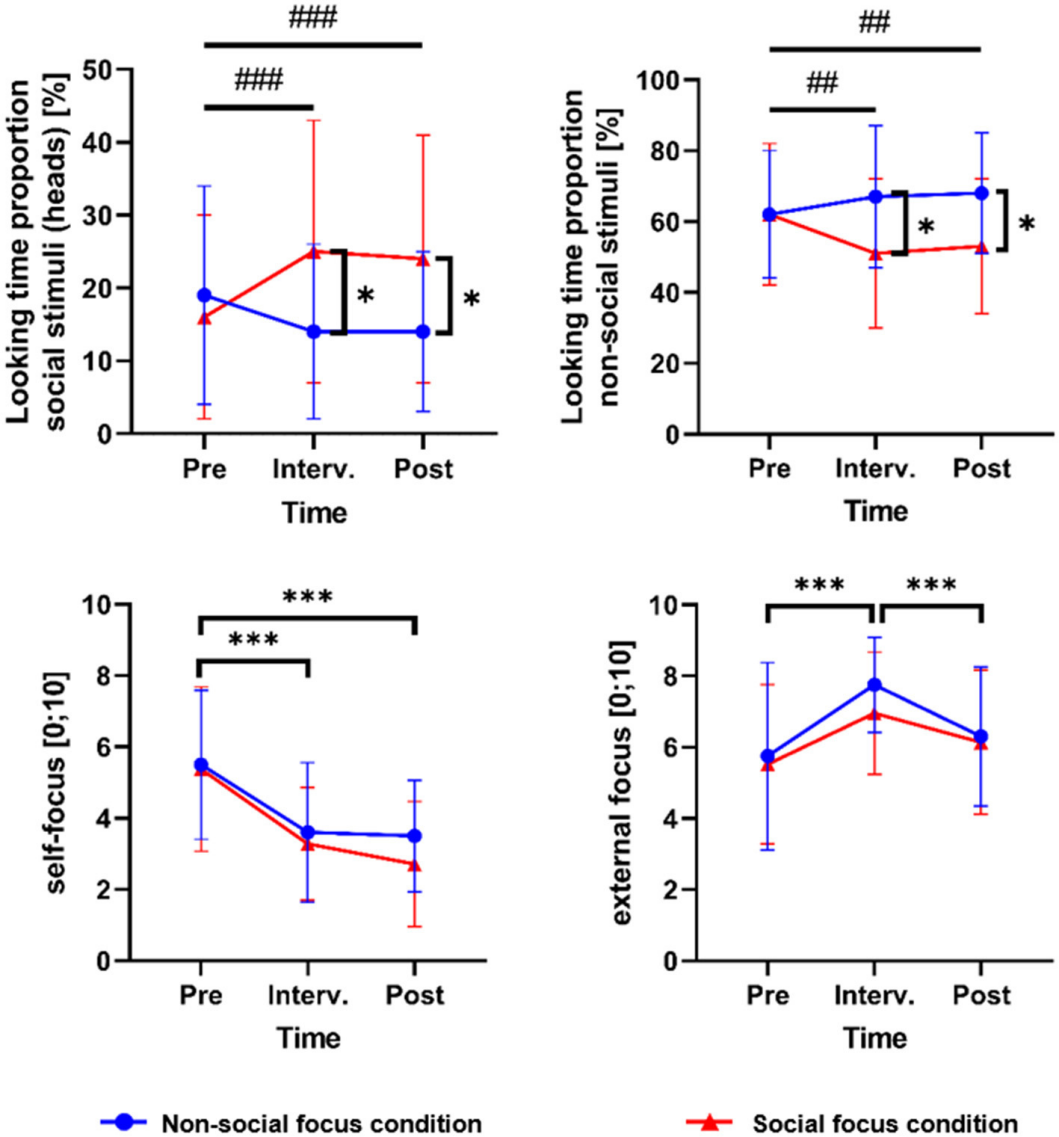
Changes in attentional processes during public speaking over all speech tasks separately for both training groups. Means (indicated by circle and triangle symbols) and standard deviations (indicated by error bars) for attention-related state outcome variables during the speech tasks at pretest, intervention, and posttest, separately for the nonsocial-focus group (*n* = 20) and the social-focus group (*n* = 21). Vertical square brackets with asterisks indicate significant group differences at pretest, intervention, or posttest (as indicated by independent-sample *t*-tests; Supplementary Table 3). Horizontal square brackets with asterisks indicate significant main effects of time between pretest and intervention, intervention and posttest, or pretest and posttest, and lines with hashtags indicate significant time × group interaction effects between the respective time points (as indicated by repeated-measures ANOVAs; Table 2 and Supplementary Table 2). */#*p* ≤ 0.050; **/##*p* ≤ 0.010; ***/###*p* ≤ 0.001. One missing value for both looking time proportion variables in the nonsocial-focus group, *n* = 19. Numeric data on mean values and standard deviations are displayed in Supplementary Table 1.

Table 2 shows mean values and standard deviations for the outcome variables at pretest and posttest separately for both experimental groups, as well as time effects and time × group interactions. Figures 2, 3 visualize changes in state variables over time separately for both groups, including measures related to the pretest, interventional, and posttest speech tasks.

**Table 2:** Changes from pretest to posttest and differences between the training groups.

	**Pre**				**Post**				**Time effects**			**Time** **×** **group interaction effects**		
	**Nonsocial focus (*****n*** **=** **20)**		**Social focus (*****n*** **=** **21)**		**Nonsocial focus (*****n*** **=** **20)**		**Social focus (*****n*** **=** **21)**							
**Variables**	***M***	***SD***	***M***	***SD***	***M***	***SD***	***M***	***SD***	***F***	***p***	** $\eta_{\text{p}}^{2}$ **	***F***	***p***	** $\eta_{\text{p}}^{2}$ **
**Affective reactions and self-perception during public speaking**														
Subjective anxiety (SUD)	36.28	22.91	45.93	28.71	17.33	13.66	23.42	18.37	74.36	**<0.001**	0.656	0.55	0.462	0.014
State anxiety (STAI state)	50.95	9.75	57.05	9.32	39.12	7.11	41.62	9.59	83.80	**<0.001**	0.682	1.46	0.234	0.036
Positive affect (PANAS)	2.81	0.77	2.43	0.69	2.85	0.69	2.86	0.91	7.73	**0.008**	0.165	5.57	**0.023**	0.125
Negative affect (PANAS)	1.79	0.70	1.96	0.71	1.24	0.41	1.30	0.35	41.81	**<0.001**	0.517	0.36	0.553	0.009
Body sensations (BSQ)	1.67	0.43	1.94	0.58	1.29	0.21	1.33	0.24	55.21	**<0.001**	0.586	3.39	0.073	0.080
Heart rate [bpm]^a^	103.87	17.04	102.78	18.88	89.89	11.35	92.19	12.43	74.34	**<0.001**	0.668	1.41	0.243	0.037
Skin conductance level [μS]^a^	5.01	2.06	4.96	1.37	4.34	1.27	4.04	1.83	8.00	**0.008**	0.178	0.21	0.650	0.006
Self-rated effect on others (single item)	3.75	2.36	2.67	2.03	5.60	1.79	4.81	2.09	44.50	**<0.001**	0.533	0.24	0.627	0.006
Self-rated appearance (single item)^b^	3.45	2.39	2.14	1.59	5.60	1.85	4.57	2.13	55.21	**<0.001**	0.586	0.20	0.654	0.005
**Attentional processes during public speaking**														
Proportion looking time social stimuli (heads)(eye tracking)^c^	0.19	0.15	0.16	0.14	0.14	0.11	0.24	0.17	0.27	0.608	0.007	21.30	**<0.001**	0.359
Self-focus (single item)	5.50	2.09	5.38	2.31	3.50	1.57	2.71	1.76	47.12	**<0.001**	0.547	0.96	0.333	0.024
External focus (single item)	5.75	2.63	5.52	2.23	6.30	1.95	6.14	2.03	3.55	0.067	0.083	0.01	0.912	0.000
**General social anxiety**														
Fear of negative evaluation (BFNE)^d^	42.60	5.85	46.52	7.33	39.95	7.39	45.43	8.10	6.83	**0.013**	0.149	1.18	0.285	0.029
Fear of public speaking (PRCS)	15.53	5.21	20.06	6.76	15.65	5.70	18.17	7.19	2.97	0.093	0.071	3.85	0.057	0.090

*The table displays mean values (M) and standard deviations (SD) for the primary (underlined) and secondary outcome variables for affective reactions and self-perception during pubic speaking, attentional processes during pubic speaking, and general Social Anxiety at pretest and posttest for participants from the nonsocial-focus condition and the social-focus condition. It further displays F-values, p-values, and $\eta_{p}^{2}$ as effect size measure for time effects from pretest to posttest and for time × group interaction effects in the outcome variables, both from repeated-measures ANOVAs. Significant p-values (≤0.050) are written in bold letters. SUD, subjective units of distress, range 0–100; STAI state, State-Trait Anxiety Inventory, range state anxiety score 20–80; PANAS, Brief Measure of Positive and Negative Affect Schedule, range subscores positive/negative affect 1–5, each; BSQ, Body Sensations Questionnaire, range total score 1–5; Self-rated effect on others/self-rated appearance/self-focus/external focus, single item score 0–10, each; BFNE, Brief Fear of Negative Evaluation Scale-Revised, range total score 12–60. PRCS, Personal Report of Confidence as a Speaker, range total score 0–30*.

a *One missing value within physiological variables in the nonsocial-focus group (n =19) and one in the social-focus group (n = 20)*.

b *Significant main effect of group for self-rated appearance, F = 4.59, p = 0.039*.

c *One missing value within eye-tracking variables in the nonsocial-focus group (n = 19)*.

d *Significant main effect of group for BFNE, F = 4.82, p = 0.034*.

#### Affective Reactions and Self-Perception

The subjective level of anxiety as a primary outcome significantly decreased in participants from both groups (main effect of time), with no significant difference in the degree of reduction between groups (time × group interaction effect) (Table 2 and Figure 2). Also, state anxiety, negative affect, bodily symptoms, heart rate, and skin conductance significantly decreased, and positive affect, self-rated effect, and self-rated appearance significantly increased over time in both groups. All pretest to posttest changes among both training groups reached large effect sizes. For positive affect, a significantly stronger increase was found in the group focusing on social stimuli during the training intervention in comparison to the group focusing on nonsocial stimuli. Dependent-sample *t*-tests showed a significant increase in positive affect during public speaking from pretest to posttest in the social-focus group (mean difference = −0.43, *t* = −2.97, *p* = 0.008) and no significant change in the nonsocial-focus group (mean difference = −0.04, *t* = −0.45, *p* = 0.660). However, indicated by an independent-sample *t*-test, the level of positive affect did not differ significantly between groups at posttest (Supplementary Table 3), which is explainable by (nonsignificant) pretest differences between groups (Table 2 and Supplementary Table 3). For all other variables besides positive affect, no significant time × group interaction effects were found, but the results showed a trend toward a stronger reduction of body sensations in the social-focus group in comparison to the nonsocial-focus group (Table 2 and Figure 2). An exploratory dependent-sample *t*-test showed a significant decrease in body sensations in the social-focus group (mean difference = 0.62, *t* = 5.37, *p* < 0.001) and also in the nonsocial-focus group (mean difference = 0.37, *t* = 5.87, *p* < 0.001), with independent sample *t*-tests revealing no significant difference between groups at posttest, but a trend toward a significant difference at pretest (Supplementary Table 3).

#### Attentional Processes

The proportion of looking time at the audience members' head as the primary outcome for attentional processes during public speaking showed no significant main effect of time from pretest to posttest, but a significant time × group interaction effect, reaching a large effect size (Table 2 and Figure 3). An independent sample *t*-test showed a significant group difference in the participants' attention toward the audience members heads at posttest, with a higher proportion of looking time in the group being instructed to focus on social stimuli during the training intervention (Supplementary Table 3 and Table 2). A dependent-sample *t*-test for the group focusing on social stimuli during the training intervention revealed a significantly higher proportion of looking time on the audience members' heads at the posttest talk in comparison to the pretest talk (mean difference = −0.07, *t* = −3.80, *p* = 0.001). In contrast, there was a significantly lower proportion of looking time on heads at posttest than at pretest in the nonsocial-focus group (mean difference = 0.06, *t* = 2.76, *p* = 0.013). An additional analysis of the participants' proportion of looking time at the audience members' whole bodies (Supplementary Table 1) also showed a significant time × group interaction effect (Supplementary Table 2), supporting the results for attention toward the audience members' heads. Furthermore, we additionally present results on pretest to posttest changes in attention toward audience members of different sexes and with positive or negative emotional expressions (Supplementary Text 3 and Supplementary Figure 1), and in attention toward nonsocial stimuli (Supplementary Text 4 and Figure 3). Self-rated internal attention as a secondary outcome for attentional processes during public speaking significantly decreased in both groups, reaching a large effect size, with no significant time × group interaction effect (Table 2 and Figure 3). Self-rated external attention as a further secondary outcome showed no significant main effect of time, but a trend toward one, and no significant time × group effect (Table 2 and Figure 3).

#### General Social Anxiety

The participants' fear of negative evaluation as a primary outcome for general Social Anxiety significantly decreased from pretest to posttest in both groups (main effect of time), reaching a large effect size, with no significant difference in the degree of reduction between groups (time × group interaction effect) (Table 2). For fear of public speaking, no significant time or time × group effect was found. Since time and time × group effects showed a tendency, exploratory analyses were conducted. Dependent-sample *t*-tests showed a significant reduction of fear of public speaking from pretest to posttest in the social-focus group (mean difference = 1.89, *t* = 2.68, *p* = 0.014), while no significant change was found in the nonsocial-focus group (mean difference = −0.12, *t* = −0.17, *p* = 0.871). However, an independent-sample *t*-test showed no significant group difference at posttest, explainable by a significantly higher pretest value in the social-focus group (Supplementary Table 3).

### Ancillary Analyses: Attention Focus During the Interventional Speech Task (Manipulation Check)

Within the VR attention focus training, the participants received the instruction to direct their attention away from self-focus to external stimuli and, according to their group assignment, specifically to either nonsocial or social external stimuli. Results from repeated-measures ANOVAs showed that self-attention significantly decreased and externally focused attention significantly increased from pretest to the intervention over both groups, with no significant time × group interaction effect (Supplementary Table 2).

Furthermore, the eye-tracking outcomes showed significant differences concerning changes in the targets of external attention between both groups. Regarding the training instruction for the nonsocial-focus group to focus on objects in the room during the interventional talk, we found a significant time × group interaction effect for the proportion of looking time on nonsocial stimuli during the interventional compared to the pretest speech task (Supplementary Table 2). Independent-sample *t*-tests revealed a significantly higher proportion of looking time on nonsocial stimuli during the intervention in the nonsocial-focus group in comparison to the social-focus group (Supplementary Tables 1, 3 and Figure 3 for mean values). Dependent-sample *t*-test showed a significant decrease in the social-focus group (mean difference = 0.10, *t* = 4.14, *p* = 0.001) and no significant change in the nonsocial-focus group (mean difference = −0.04, *t* = −1.06, *p* = 0.304). However, no change does not stand in contrast to the instruction of the nonsocial-focus condition to focus on objects in the room during the intervention. A high proportion of looking time on nonsocial stimuli was already found in the nonsocial-focus group during the pretest speech task (*M* = 0.62, *SD* = 0.19) and seemed to be maintained or even slightly increased during the interventional speech task (*M* = 0.67, *SD* = 0.20) (Supplementary Table 1).

Regarding the training instruction in the social-focus group to focus on the audience members' faces during the interventional talk, a significant time × group interaction effect was found for the proportion of looking time at the audience members' heads during the interventional compared to the pretest speech task (Supplementary Table 2). Independent-sample *t*-tests revealed a significantly higher proportion of looking time on the audience members' heads during the intervention in the social-focus group in comparison to the nonsocial-focus group (Supplementary Tables 1, 3 and Figure 3 for mean values). Dependent-sample *t*-tests showed a significant increase in the participants' attention at the audience members' heads from pretest to the intervention in the social-focus group (mean difference = −0.09, *t* = −4.04, *p* = 0.001) and no significant change in the nonsocial-focus group (mean difference = 0.05, *t* = 1.69, *p* = 0.108). An additional analysis of changes between the pretest and the interventional speech task in attention toward the heads of audience members of different sexes and with different emotional expressions sitting in the first row is reported in Supplementary Text 1 and visualized in Supplementary Figure 1.

## Discussion

### Summary of Main Results

This randomized controlled study examined the effects of two versions of a one-session VR attention training for Social Anxiety, combining virtual exposure to public speaking with an attentional shift from self-focus to either nonsocial or social external stimuli. As novel aspects, we used the possibilities of VR to create a virtual training environment and examined differential effects of a focus on nonsocial vs. social external stimuli when directing the attention away from self-focus during public speaking. A manipulation check showed that participants followed the attentional instructions during the public speaking exposure, indicated by a significant reduction of self-focus and increase in external focus during the training intervention compared to a pretest speech task, a significant increase in the proportion of looking time toward social stimuli in the social-focus group, and a maintenance of a high proportion of looking time on objects in the nonsocial-focus group.

From before to after the attention training, a significant decrease of subjective anxiety during public speaking and fear of negative evaluation as primary outcomes was reached in participants from both groups, with no significant differences in the reduction depending on the specific external-focus instruction. For the attention toward the audience members' heads as a further primary outcome, a significantly different change was found in the social-focus group in comparison to the nonsocial-focus groups. The proportion of looking time at the listeners' heads during giving a talk significantly increased from before to after the intervention in the social-focus group, whereas it significantly decreased in the nonsocial-focus group. Therefore, the hypothesis of an advantage of shifting the attentional focus from the self to social stimuli instead of nonsocial stimuli during an attention focus training cannot be accepted for subjective anxiety during public speaking and fear of negative evaluation but can be accepted for the participants' proportion of looking time at the audience members' heads. Accordingly, the target of external attention differently changed depending on experimental condition, and this change was maintained up to the posttest according to our hypothesis.

For the secondary outcomes state anxiety, positive affect, negative affect, body sensations, heart rate, skin conductance, self-focused attention, self-rated effect on others, and self-rated appearance in front of others, significant improvements from before to after the intervention were found in participants from both training groups. External attention and fear of public speaking, though, did not change significantly within both groups but showed trends toward improvements. Further differential effects between the social- and the nonsocial-focus groups were only found for positive affect, with an increase from before to after the intervention only within the group focusing on social stimuli during the attention training. Furthermore, for body sensations and fear of public speaking, a trendwise different change was found in the social-focus group in comparison to the nonsocial-focus group, but higher pretest values in the social-focus group changing into posttest values that were not significantly different between groups impede the interpretation. However, while body sensations significantly decreased in both groups, fear of public speaking only decreased in the social-focus group, pointing to a different development of fear of public speaking in participants with a social vs. nonsocial focus during the training intervention.

### Interpretation

Previous attention trainings for Social Anxiety aiming at a reduction of self-focused attention conducted attention flexibilization trainings outside socially stressful situations (51–53), or trained participants to focus on the task itself (54, 55) or on external cues (56–58) also within socially stressful situations. Our attentional training is related to the last category but is conducted in VR for the first time. Previous studies showed that external-focus training is an effective intervention and partially more effective than other interventions for Social Anxiety, e.g., concerning negative self-thoughts when compared to a positive self-imagery intervention (57) or concerning a shift from the observer to the field perspectives and from self-focus to external focus and concerning anxiety and negative beliefs when compared to plain exposure (58) (see also Introduction). In line with positive results from those foregoing studies, our external-focus attention training yielded broad improvements among participants from both training versions concerning self-focus, subjective anxiety, state anxiety, positive affect, negative affect, body sensations, heart rate, skin conductance, self-rated effect, and self-rated appearance during public speaking, as well as fear of negative evaluation. This provides the first evidence that a training to reduce self-focused attention by increasing external focus can be successfully conducted in VR. However, previous studies included the instruction to focus externally on other people (57, 58) but did not examine differential effects of an external social vs. nonsocial focus as a strategy to reduce self-focused attention. Therefore, our results provide novel findings concerning different targets of external attention.

As a key finding concerning differential effects, our results show that only a focus on social stimuli during the attention training but not a focus on nonsocial stimuli resulted in an improvement of attention toward the audience members' heads (representing eye contact) from before to after the intervention. Overt attention to the audience's faces can be considered a desirable outcome for a public speaking training. First, attention toward the audience members is regarded as beneficial in regard to a disconfirmation of dysfunctional beliefs (11). Therefore, an increase might be advantageous for coping with Social Anxiety. Second, eye contact can be regarded as a quality aspect of the speaking performance. Within cognitive models, performance deficits due to anxiety (11, 12, 15, 16) or due to a lack of social skills or knowledge (12, 15, 16) are considered as a maintaining factor for Social Anxiety, e.g., by supporting a negative mental representation of the self as seen by others through negative self or external social evaluation. Thus, the increased eye contact to the audience in participants from the social-focus group might elicit a more positive self-evaluation of one's own speaking performance and promote positive external feedback during real-life speeches in the future. In this regard, follow-up tests on giving talks in real-life are needed, and future studies should examine the participants' behavior during speech tasks also from a listener's perspective.

As further advantage, participants of the social-focus condition showed a significant increase in positive affect during public speaking from before to after the intervention, while no significant change was found in participants from the nonsocial-focus group. One might speculate that participants exhibit a more positive speaking experience after having confronted themselves with social stimuli by directing attention toward the audience. However, the interpretation of the significant interaction effect is impeded by posttest values not significantly different between groups (see Results). Further studies are demanded to confirm our findings of stronger increases in positive affect during and after a social focus. One important question here would be whether the emotional display of the focused audience might influence the affect and further coping with the situation.

Besides improved attention toward the audience and indications for improved positive affect during public speaking, no further clear advantages were found for a social focus in comparison to a nonsocial focus during the attention training. Instead, both training versions of directing the attentional focus away from the self to either social or nonsocial external stimuli resulted in broad improvements concerning affective reactions and self-perception during public speaking, as well as general fear of negative evaluation. This stands in contrast to the hypothesis that a nonsocial focus might work as a distraction, avoidance, or safety behavior impeding the reduction of Social Anxiety (11) and demands further discussion.

As first consideration, the improvements among both versions of external focus could be drawn back to a decrease in self-focus reached in both training groups. Referring to Wells et al. (58), shifting the attention away from anxiety symptoms and their intensity by focusing externally reduces the activation of negative self-images. This argumentation is supported by the large improvements in the self-rated effect on others and self-rated appearance in front of others among participants from both of our training groups. The participants' perception of their own performance as a speaker is regarded as one aspect of their self-images during public speaking (11, 12, 15). It might be speculated that in our intervention the change of attention from a self-focus to external stimuli is much more relevant for the emotional experience than the specific target of the external focus.

As a second consideration, distraction from social stimuli might be specifically helpful during public speaking. Regarding anxiety levels during exposure, Craske et al. (90) compared focused and distracted exposure for specific phobia and found increasing subjective fear during the focused exposure condition, but stable fear levels during the distracted exposure and a natural exposure condition, and no differences for heart rate. Moreover, for a one-session VR exposure treatment in flight phobia, breathing exercises during exposure were associated with lower fear and trendwise larger treatment outcome compared to attention focus on fear stimuli (91). Regarding effects of distraction and safety behaviors during exposure on anxiety levels after exposure, in contrast, Grayson et al. (92) found that participants with obsessive-compulsive disorder who had distracted their attention during exposure showed a greater return of fear at the follow-up measurement, while participants who had directed their attention toward the feared stimulus showed a remaining reduction of fear. However, a replication study using the same protocol (93) found contradictory results. For social-evaluative situations, a review by Wong and Rapee (16) on experimental studies showed that exposure without behavioral avoidance or escape resulted in a decrease of Social Anxiety and further positive social outcomes and that using safety behaviors led to higher, and its reduction to lower, social-evaluative anxiety and self-judgements. In general, a large body of research on this topic exists, mainly for specific phobia, and reviews summarizing those studies could not show consistent results (16, 94, 95). For specific phobia, evidence exists that judicious use of safety behavior might not reduce or even enhance the effect of exposure, when mastery experiences are made and safety behavior is reduced between sessions (95–97). As summarized in the Introduction section, another category of attention trainings for Social Anxiety aims at a disengagement from social threat cues instead of promoting attention toward them and guided participants to direct their attention to nonthreatening aspects of their social environment (18). Reviews and meta-analyses (39, 40, 42, 43) as well as recent studies on VR-based trainings (44, 45) provide contradictory results concerning the effects on Social Anxiety. However, since mainly early responses (500 to 1,000 ms) toward visual or word stimuli were trained in computerized interventions in order to reduce initial hypervigilance, the results seem not applicable to attention focus modulation during a public speaking exposure lasting several minutes. After all, no previous study on Social Anxiety specifically investigated the effects of a reduction of self-focused attention during a socially threatening situation by visually engaging with social stimuli or visually disengaging with social stimuli by focusing on nonsocial stimuli. Our results show reduced anxiety during the interventional speech task as well as during the posttest speech task conducted directly after the intervention within both groups, while the respective external-focus instruction during training was further maintained at posttest within both groups although no longer instructed (Figure 3 and Supplementary Text 5). Future studies are demanded to examine mid- and long-term effects of our social- vs. nonsocial-focus attention training and to disentangle positive short-term effects of distraction during exposure and follow-up effects concerning the maintenance of Social Anxiety.

This leads to the third consideration that potential advantages of the social-focus training might first be revealed on the long run, e.g., by promoting the disconfirmation of beliefs through an occupation with social stimuli. Directing attention to the audience members provides the possibility to process information about their actual behavior and thereby to check and correct dysfunctional beliefs and appraisals about social evaluation. Furthermore, the self-perception of holding visual contact to the audience, considered as a positive speaking behavior, might further improve the participants' mental representation of their self as seen by the audience. Also Clark and Wells (11) and Wells et al. (51) proposed that attentional strategies are effective in the long term by modifying dysfunctional appraisals and restructuring maladaptive processing configurations. They refer to attention strategies instructing patients to direct their attention toward information disconfirming negative beliefs and appraisals, e.g., interrogate features of the external environment instead of self-focus. At the same time, they warn against the use of distraction by neutral stimuli within a social situation, because this might deplete attention needed for the disconformity processing, and the fact that a catastrophe is not happening could be attributed to the use of those strategies instead of the actual likelihood of a catastrophe. As potential indication for an advantage of our social-focus training concerning the reduction of Social Anxiety, improvements in positive affect during public speaking were found only in the social-focus group but not in the nonsocial-focus group. As further potential indication, no significant time but a trendwise interaction effect was found for the participants' general fear of public speaking, with a significant reduction from before to after the intervention in the social-focus group, and no significant change in the nonsocial-focus group. It might be a methodical problem of our study that the time point of the posttest measurement has been too early to fully capture improvements and differences in improvements between the training interventions. For example, the assessment of fear of public speaking and fear of negative evaluation were both conducted after the interventional speech task, but before the posttest speech task. Therefore, participants might have been still standing under the experience of the exposure toward social or respectively, nonsocial cues during the training intervention. Post-event processing concerning their beliefs might still take place at this time point, and the participants had not yet had the chance to experience themselves in a public speaking situation again. Furthermore, a repeated observance of the audience members' reactions, also in real-life situations, might be necessary to fully realize a disconfirmation of dysfunctional beliefs about negative social evaluation, or to receive positive feedback for an improved speaking performance in regard to eye contact. Future studies on mid- and long-term effects of our social- and nonsocial-focus attention training would provide the possibility to capture the advantages of the social stimuli version not assessable within the current study design.

As a fourth consideration, the necessity of specifically promoting habituation or disconfirmation of dysfunctional beliefs to reveal stronger advantages for the social-focus training can be discussed. Although providing the possibility to disconfirm maladaptive beliefs and appraisals concerning the audience by intensively focusing on them during the interventional speech task in the social-focus group, the participants were not explicitly instructed to check whether expected catastrophes actually appeared. Furthermore, the interventional speech task with attention modulation was not constructed to reach habituation to the social cues in all participants from the social-focus group. Due to the fixed time period of 5 min for the interventional speech task, the task might have been finished before reaching a certain degree of habituation in some participants. Therefore, stronger effects of the social-focus condition might be expected by an individualized length of the interventional speech task. However, the latest theories do not consider a fear reduction as necessary for a successful exposure any more but rather argue that processes of inhibitory learning constitute efficacious treatments (98, 99). In CBT for Social Anxiety Disorder and the underlying Cognitive Model of Social Phobia (11), aspects like belief disconfirmation and omitting safety-seeking behaviors are regarded as more essential than prolonged exposure (100, 101). Our attention modulation training did not include cognitive interventions on the explicit verification and correction of dysfunctional beliefs and appraisals concerning social evaluation after confrontation participants from the social-focus group with the audience members' reactions during the interventional speech task. A previous study on external-focus training by Wells et al. (58), in contrast, specifically instructed participants to look around and observe other people closely in order to discover their reaction and check out if their own fears are true. The significant advantages concerning a decrease of anxiety and negative beliefs in comparison to plain exposure within this study might point to the relevance of an external-focus instruction including information on the importance of disconfirming negative beliefs and appraisals. In future, our VR attention training could be combined with interventions for conscious disconformity processing, possibly being then able to exhaust the full potential of focusing on social stimuli during the intervention.

As a fifth consideration, the relevance of attention toward or avoidance of external positive or external negative social stimuli for the disconfirmation of dysfunctional beliefs and appraisals must be discussed. Interestingly, during the training intervention, the participants in the social-focus group increased their attention toward the two male audience members with one positive and one negative emotional expression and to the female audience member with a positive expression, while no significant change in attention was found toward the female with a negative expression (Supplementary Text 1). The female auditory member showing the negative expression was rated the most unfriendly and annoyed among all other audience members, also in comparison to the male audience member showing the negative expression (Supplementary Table 4). Therefore, the finding might indicate that despite the social-focus instruction, participants from the social-focus group mainly avoided looking at the most negatively valent audience member. Alternatively, the negative female audience member was sitting at the outer right position, and therefore, the virtual agent's position in the visual environment might have influenced attentional processes. Referring to previous studies, an avoidance of socially threatening audience members is in line with the work of Rubin et al. (29), examining gaze behavior during public speaking in individuals with Social Anxiety. They found that individuals with higher fear of public speaking showed stronger avoidance of uninterested audience members in comparison to interested audience members. Concerning the effects of our social-focus training, it could be speculated that the social focus training might show stronger advantages if attention also toward external negative social cues would be specifically promoted. Coming from this question, future studies should systematically examine differences between an attention training instructing an external social focus on positively valent vs. negatively valent social stimuli during public speaking.

Finally, it is to consider that the nonsocial-focus training in comparison to the social-focus training did not generally lead to lower levels of anxiety during the interventional public speaking exposure with attention modulation instruction. Therefore, the social stimulus focus version of our attention focus modulation intervention must not be considered as more burdensome for participants than the nonsocial-focus condition, which could help to motivate patients for an exposure toward social stimuli during an interventional speech task, especially if more advantages of the social-focus version might be confirmed in future studies on long-term effects.

As additional point for discussion, we did not find significant pretest to posttest improvements in external attention among both groups. The variable only showed a trendwise increase over both groups, and no significant interaction effect pointing to an increase in one but not in the other group. Wells et al. (58), in contrast, found a higher shift from an observer to a field perspective and from self-focus to external focus when comparing exposure plus an external-focus instruction with plain exposure. Similar to our results, Woody et al. (56) found a decrease in self-focus but no increase in external focus over the course of a CBT with external-focus training. The authors suggest that information-processing theories should be modified in regard to their proportional view of internal and external focus of attention but also considered lacking validity of the measurements for self-focus and external focus. In this regard, future studies could include biological correlates of self-focus and external focus as additional measures besides self-ratings. A study by Boehme et al. (102) showed a hyperactivated medial prefrontal cortex, temporoparietal junction, and temporal pole during inward-focused attention in comparison to outward-focused attention in highly socially anxious participants compared to low socially anxious participants. Furthermore, higher activation of medial prefrontal cortex, right anterior insula, temporoparietal junction, and posterior cingulate cortex was found to be associated with higher trait values of self-focused attention in highly socially anxious subjects. As further interpretation of our results, the significant increase in self-rated external focus from before to during the training intervention (see Ancillary analyses), and the significant decrease from the intervention to posttest within both groups of our study (Supplementary Text 5; Figure 3 and Supplementary Table 2) might indicate that participants failed in maintaining (the perception of) an extent of external focus without an explicit instruction. Future applications of the training might introduce booster instructions to maintain a high level of external focus also after the training intervention.

## Limitations

As the first limitation, we examined participants with high levels of Social Anxiety indicated by a SPIN score ≥19, but only five participants fulfilled the criteria for a Social Phobia Disorder according to DSM-criteria, as indicated by the M.I.N.I. interview. However, we used a SPIN cutoff score validated to screen for Social Phobia (60), providing effects for highly socially anxious participants that might be transferrable also to patients with Social Phobia. Future studies should additionally examine the training effects in clinical samples with Social Anxiety Disorder, which actively search treatment and with mental disorder comorbidities like depressive disorders. Secondly, as mainly female and younger participants were included, the generalizability on male, as well as on older persons or children and adolescents, must be examined in further studies. Thirdly, the interpretation of results for particular variables is complicated by pretest group differences for state anxiety (STAI state), self-rated appearance, and fear of public speaking (PRCS) (Supplementary Table 3). However, repeated-measures ANOVAs were chosen as statistical analysis for detection of training effects, controlling for these pretest differences. Moreover, baseline differences always can appear after randomization and should not be over-interpreted, especially since they have not been expected, and thus, no hypothesis has been formulated on such differences. However, the study could be replicated in larger samples for a confirmation of the results. Fourth, only single items have been used for the assessment of the participants' self-rated internal focus, external focus, effect on others, and appearance in front of others, due to time efficiency and a lack of established German questionnaires on those constructs. Fifth, the accuracy of the eye-tracking system and the resulting interpretation of gaze with regard to the virtual environment was not evaluated systematically. It is possible that for some participants, errors in measurement resulted in not registering the correct AOI. Adding extended shapes around the body and head of agents might have resulted in more accurate measurement, but we do not think it would have substantially changed our eye-tracking results. Sixth, the analyses of proportions of looking time on regions of interest can cause certain problems in the areas of high and low values, and therefore, transformations are recommended (103). As such transformations were not eligible for our data due to their distribution, all analyses for eye-tracking-related outcomes were additionally conducted for the dwell time as a further outcome variable (Supplementary Tables 1, 2). The problem with dwell time data is a different amount of missing data between participants, but they provide additional information. Seventh, as the task was focused on giving a talk in front of an audience, we cannot draw conclusions on different social situations relevant in Social Anxiety, e.g., eating in front of others, starting to speak to someone, and claiming one's own rights. Future studies could examine the effects of the VR attention modulation training adapted for use in different social situations. In this regard, the transferability of the general training effects into real-life social situations must also be further examined. Eighth, as there was no control group with plain exposure to public speaking without an attention focus training, improvements among both groups cannot be clearly attributed to the attention focus modulation but might be attributable to the exposure to public speaking in front of an audience. However, the manipulation check showed that the participants mainly followed the training instruction during the interventional speech task, and as an internal reference strategy, the significant decrease in self-focused attention from pretest to posttest in both groups indicates a specific outcome of the attention training not to be suspected within a plain exposure treatment. Furthermore, a previous study compared exposure plus external attention focus instructions with plain exposure and showed significantly stronger effects of the additional attention instruction concerning the reduction of within-situation anxiety and beliefs in feared catastrophes (58). Lastly, the lack of follow-up measures impedes conclusions on mid- and long-term effects of the training, which should be examined in future studies. Especially for variables on general Social Anxiety, an assessment at least after the posttest speech task should be realized.

## Conclusion

This randomized controlled study examined the differential effects of two versions of a VR attention focus training for Social Anxiety, instructing participants to reduce self-focused attention by shifting their attentional focus to either social or nonsocial external stimuli during exposure to public speaking. Both training versions showed positive pretest to posttest effects in highly socially anxious participants, suggesting that an external attention focus training can be successfully implemented as a one-session VR intervention and that a nonsocial focus during exposure to public speaking does not impede the pretest to posttest reduction of anxiety. However, focusing on social stimuli during the training yielded significant advantages for public speaking after the intervention, in the form of an improvement in overt attention to the audience members' heads. There are also indications that only a social focus might result in improvements concerning positive affect during public speaking and fear of public speaking, but further confirmation is needed. Changes in overt attention toward social stimuli might enforce corrective cognitive processing of relevant social stimuli and represent an improvement in the speaking performance and thereby result in even broader advantages of a social focus in the middle and long term. Future research should examine later effects of the social-stimuli-directed and the nonsocial-stimuli-directed versions of the VR attention focus modification training including effects in external ratings of the speaking performance and effects on real-life social situations, e.g., to be examined under usage of portable eye-tracking glasses. Furthermore, combining an attentional shift toward social stimuli with cognitive interventions to explicitly support cognitive reinterpretations of dysfunctional beliefs and appraisals might provide advantages and should be examined in the future. In addition, feedback for the participants concerning their attentional processes measured via eye tracking might be an interesting extension. In general, VR tasks and their combination with eye tracking could be used fruitfully to further investigate the role of the content of different kinds of external social focus and to guide patients to regulate attention processes during exposure interventions in treating Social Anxiety.

## Data Availability Statement

The raw data supporting the conclusions of this article will be made available by the authors, without undue reservation.

## Ethics Statement

The studies involving human participants were reviewed and approved by Ethikkommission bei der Universität Regenburg Universität Regensburg 93040 Regensburg ethikkommission@ur.de. The patients/participants provided their written informed consent to participate in this study.

## Author Contributions

TW had the idea for the research question. TW, MP, RE, and AM developed the study design, materials and methods, and procedure. RE and LS recruited the participants. RE, LS, and TW were responsible for the data collection. TW conducted the data preprocessing and statistical analyses under contributions of MP, RE, LS, and AM. MP was responsible for the processing and analyses of the eye-tracking data in specific. TW drafted the manuscript under consultation of AM. All authors contributed to and critically reviewed the final draft and agreed to be accountable for the content of the work.

## Conflict of Interest

AM is a stakeholder of a commercial company that develops virtual environment research systems. The remaining authors declare that the research was conducted in the absence of any commercial or financial relationships that could be construed as a potential conflict of interest.

## Publisher's Note

All claims expressed in this article are solely those of the authors and do not necessarily represent those of their affiliated organizations, or those of the publisher, the editors and the reviewers. Any product that may be evaluated in this article, or claim that may be made by its manufacturer, is not guaranteed or endorsed by the publisher.
