# Supplementary material for: Look at the Audience? A Randomized Controlled Study of Shifting Attention From Self-Focus to Nonsocial vs. Social External Stimuli During Virtual Reality Exposure to Public Speaking in Social Anxiety
Source: Front Psychiatry. 2021 Dec 14;12:751272. doi: 10.3389/fpsyt.2021.751272 (PMC8712494; doi:10.3389/fpsyt.2021.751272)
Supplement: Supplementary file 1 [file Data_Sheet_1.docx]

# Supplementary Material

## Supplementary Text

### Supplementary Text 1 │ Attention towards female and male audience members with positive and negative emotional expressions during the training intervention.

Concerning attention towards the heads of audience members of different sexes and with negative and positive expressions sitting in the first row (Figure 1B), there was a significant time x group interaction effect from pretest to the intervention for the proportion of looking time on the head of the male and female showing a positive expression, and the male showing a negative expression (Supplementary Table 2). Dependent sample *t*-tests indicate a significant increase in the social focus group (male positive: mean difference=–0.04, *t*=–4.38, *p*=<0.001; male negative: –0.02, *t*=–2.41, *p*=0.026; female positive: mean difference=–0.02, *t*=–2.34, *p*=0.030), but no significant change in the nonsocial focus group (male positive: mean difference=–0.00, *t*=–0.45, *p*=0.661; male negative: 0.02, *t*=1.96, *p*=.066; female positive: mean difference=0.01, *t*=0.93, *p*=0.365) (Supplementary Figure 1 and Supplementary Table 1 for mean values). Independent sample *t*-test showed a significantly higher proportion of looking time on the positive female audience members’ head during the interventional speech task in the social focus in comparison to the nonsocial focus group, and by trend higher values for the positive and negative male audience members’ heads (Supplementary Table 3). For the looking time proportion at the negative female member’s head, no significant time x group effect was found (Supplementary Table 2), and also dependent sample *t*-tests showed no significant changes in the social focus group, mean difference=–0.00, *t*=–0.56, *p*=0.585, and in the nonsocial focus group, mean difference=0.00, *t*=1.05, *p*=0.308. For the participants’ ratings of the perceived valence of the audience members’ expressions see Supplementary Table 4.

### Supplementary Text 2 │ Affective reactions and self-perception during the training intervention.

We additionally examined differences in the participants’ affective responses and self-perception during the two different versions of the VR attention focus training. Results from repeated-measures ANOVAs showed significant changes from the pretest to the interventional speech task over both groups for ratings of anxiety, state anxiety, negative affect, bodily symptoms, heart rate, self-rated effect on others, and self-rated appearance, but not for positive affect and skin conductance level (Supplementary Table 2 and Figure 2). There were no time x group interaction effects for all variables, except for body sensations (Supplementary Table 2 and Figure 2). Within dependent sample *t*-tests, a significant decrease in bodily symptoms was found in the nonsocial focus group, mean difference=0.23, *t*=5.13, *p*=<0.001, and in the social focus group, mean difference=0.44, *t*=4.81, *p*=<0.001, and independent sample *t*-tests indicate no significant group difference during the interventional talk (Supplementary Table 3), due to a trendwise group difference at pretest with higher values in the social focus group (Supplementary Table 3; Figure 2 and Supplementary Table 1 for mean values).

### Supplementary Text 3 │ Pretest to posttest changes in attention towards female and male audience with positive and negative emotional expressions.

Additionally to analyzing pretest to posttest changes in the proportion of looking time on the audience members heads in general, we specifically analyzed these attention focus measures separately for each of the four agents sitting in the first row (Figure 1B). We found no significant time effect over both groups, but a significant time x group effect for the participants’ proportion of looking time on the heads of the male and female showing a positive expression, and the male showing a negative expression (Supplementary Table 2; Supplementary Figure 1 and Supplementary Table 1 for mean values). Dependent sample *t*-tests for the looking time proportion on the head of the male showing the positive expression indicate a significant increase from pretest to posttest in the social focus group, mean difference=–0.04, *t*=–6.29, *p*=<0.001, but not in the nonsocial focus group, mean difference=0.00, *t*=0.33, *p*=0.749. An independent sample *t*-test shows a trendwise group difference at posttest, but the interpretation is impeded due to significant pretest differences between groups (Supplementary Table 3). For the looking time proportion on the head of the male showing the negative expressions, there was a significant increase in the looking time proportion from pre- to posttest in the social focus group, mean difference=–0.02, *t*=–2.41, *p*=0.026, a significant decrease in the nonsocial focus group, mean difference=0.02, *t*=2.89, *p*=0.010, and a significant higher posttest value in the social focus group in comparison to the nonsocial focus group (Supplementary Table 3). On the positive female member’s head, there was a significant increase in the looking time proportion from pre- to posttest in the social focus group, mean difference=–0.02, *t*=–2.81, *p*=0.011, no significant change in the nonsocial focus group, mean difference=0.01, *t*=1.22, *p*=0.238, and significant higher posttest value in the social focus group in comparison to the nonsocial focus group (Supplementary Table 3). For the proportion of looking time on the female audience member with a negative emotional expression, our results showed no significant time effect from pre- to posttest, and no significant time x group effect. Interestingly, the participants’ ratings of this audience member emotional expressions descriptively showed the highest intensity of negative emotional expressions, and the lowest rated intensity of positive emotional expressions of all audience members (Supplementary Table 4).

### Supplementary Text 4 │ Pretest to posttest changes in attention towards nonsocial stimuli.

An additional analysis of the proportion of looking time on nonsocial stimuli revealed no significant time effect from pretest to posttest over both groups, but a significant time x group interaction effect (Supplementary Table 2 and Figure 3), with dependent sample *t*-test showing a significant increase from pretest to posttest in the nonsocial focus group (mean difference=–0.06, *t*=–2.17, *p*=0.043), and a significant decrease in the social focus group (mean difference=0.09, *t*=2.66, *p*=0.015); and with an independent sample *t*-test showing a significantly higher looking time proportion on nonsocial stimuli at posttest in the nonsocial focus in comparison to the social focus group (Supplementary Table 3).

### Supplementary Text 5 │ Maintenance of effects after the intervention (Changes in all state variables from the intervention to posttest).

To examine the maintenance of effects from the interventional speech task to the posttest speech task, we conducted repeated measures ANOVAs analyzing time effects in affective reactions, self-perception, and attentional processes during public speaking from the interventional speech task to the posttest speech task, and differences in those changes between groups (time x group interaction effects).

Results showed a significant change from the intervention to posttest among both groups for anxiety levels, state anxiety, positive affect, negative affect, bodily symptoms, heart rate, skin conductance level, self-rated effect on others, self-rated appearance, and external focus (Supplementary Table 2). The mean values showed an increase in positive affect, and a decrease in subjective anxiety, state anxiety, negative affect, bodily symptoms, heart rate, self-rate effect on others, and self-rated appearance within both groups, indicating further improvements between the interventional speech task and the post speech task among both groups (Supplementary Table 1, Figure 2). In contrast, for externally focused attention, a significant decrease from the intervention to posttest was found within both groups (Supplementary Table 2), indicating a decline in the direction of pretest levels (Supplementary Table 1 and Figure 3). No significant main effect of time was found for self-focused attention, the proportion of looking time on the audience members’ heads, and the proportion of looking time on nonsocial stimuli (Supplementary Table 2), indicating a stable maintenance of the levels from the intervention to posttest in both groups (Supplementary Table 1 and Figure 3). There were no significant time x group interaction effects for any variable, though, a trendwise interaction effect was found for negative affect (Supplementary Table 2). Means point to a stronger decrease from the intervention to posttest in the social focus in comparison to the nonsocial focus group (Supplementary Table 1, Figure 2).

## Supplementary Figures

Supplementary Figure 1 │ Looking time proportion on female and male audience members with positive and negative emotional expressions during public speaking over all speech tasks separately for both groups.


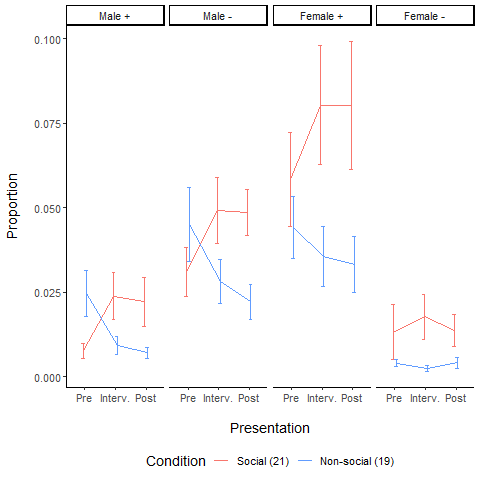


***Note.*** Looking time proportions on the audience members sitting in the first row of the virtual lecture room (Figure 1B) measured via eye tracking during the speech tasks at pretest, intervention, and posttest are displayed. One missing value for all variables in the nonsocial focus group, *n*=19.

## Supplementary Tables

### Supplementary Table 1 │ Comprehensive data for state outcome variables for the pretest, interventional, and posttest speech task.

|  | **Nonsocial condition (*n*=20)** | | | | | | **Social condition (*n*=21)** | | | | | |
| --- | --- | --- | --- | --- | --- | --- | --- | --- | --- | --- | --- | --- |
|  | **Pretest** | | **Intervention** | | **Posttest** | | **Pretest** | | **Intervention** | | **Posttest** | |
| **Variables** | *M* | *SD* | *M* | *SD* | *M* | *SD* | *M* | *SD* | *M* | *SD* | *M* | *SD* |
| Subjective anxiety (SUD) | 36.28 | 22.91 | 23.61 | 18.11 | 17.33 | 13.66 | 45.93 | 28.71 | 34.71 | 23.85 | 23.42 | 18.37 |
| State anxiety (STAI state) | 50.95 | 9.75 | 42.05 | 7.14 | 39.12 | 7.11 | 57.05 | 9.32 | 47.43 | 9.17 | 41.62 | 9.59 |
| Positive affect (PANAS) | 2.81 | 0.77 | 2.80 | 0.67 | 2.85 | 0.69 | 2.43 | 0.69 | 2.61 | 0.74 | 2.86 | 0.91 |
| Negative affect (PANAS) | 1.79 | 0.70 | 1.33 | 0.34 | 1.24 | 0.41 | 1.96 | 0.71 | 1.58 | 0.43 | 1.30 | 0.35 |
| Body sensations (BSQ) | 1.67 | 0.43 | 1.44 | 0.31 | 1.29 | 0.21 | 1.94 | 0.58 | 1.51 | 0.34 | 1.33 | 0.24 |
| Heart rate [bpm]^a^ | 103.87 | 17.04 | 94.15 | 11.10 | 89.89 | 11.35 | 102.78 | 18.88 | 95.94 | 12.47 | 92.19 | 12.43 |
| Skin conductance level [μS]^a^ | 5.01 | 2.06 | 5.34 | 2.63 | 4.34 | 1.27 | 4.96 | 1.37 | 4.67 | 1.70 | 4.04 | 1.83 |
| Self-rated effect on others (single item) | 3.75 | 2.36 | 5.10 | 1.68 | 5.60 | 1.79 | 2.67 | 2.03 | 3.95 | 1.63 | 4.81 | 2.09 |
| Self-rated appearance (single item) | 3.45 | 2.39 | 4.90 | 1.86 | 5.60 | 1.85 | 2.14 | 1.59 | 3.95 | 1.83 | 4.57 | 2.13 |
| Proportion looking time heads (eye tracking)^b^ | 0.19 | 0.15 | 0.14 | 0.12 | 0.14 | 0.11 | 0.16 | 0.14 | 0.25 | 0.18 | 0.24 | 0.17 |
| Dwell time heads (sec)^b^ | 50.60 | 41.67 | 35.24 | 32.00 | 33.87 | 33.01 | 41.17 | 38.25 | 58.15 | 48.38 | 56.86 | 41.42 |
| Proportion looking time whole bodies^b^ | 0.34 | 0.18 | 0.30 | 0.19 | 0.28 | 0.16 | 0.35 | 0.19 | 0.45 | 0.20 | 0.43 | 0.18 |
| Dwell time whole bodies (sec)^b^ | 89.28 | 49.31 | 72.44 | 50.92 | 68.76 | 43.61 | 88.49 | 56.08 | 100.94 | 62.86 | 104.95 | 50.90 |
| Proportion looking time nonsocial stimuli^b^ | 0.62 | 0.18 | 0.67 | 0.20 | 0.68 | 0.17 | 0.62 | 0.20 | 0.51 | 0.21 | 0.53 | 0.19 |
| Dwell time nonsocial stimuli (sec)^b^ | 160.54 | 51.76 | 163.15 | 55.62 | 163.86 | 48.95 | 148.47 | 53.21 | 104.45 | 50.53 | 126.20 | 49.10 |
| Proportion looking time head male-positive^b^ | 0.02 | 0.03 | 0.01 | 0.01 | 0.01 | 0.01 | 0.01 | 0.01 | 0.02 | 0.03 | 0.02 | 0.03 |
| Dwell time head male-positive (sec)^b^ | 6.57 | 8.22 | 2.26 | 3.32 | 1.75 | 2.11 | 1.89 | 2.48 | 5.06 | 5.61 | 4.89 | 7.30 |
| Proportion looking time head male-negative^b^ | 0.05 | 0.05 | 0.03 | 0.03 | 0.02 | 0.02 | 0.03 | 0.03 | 0.05 | 0.04 | 0.05 | 0.03 |
| Dwell time head male-negative (sec)^b^ | 11.98 | 13.57 | 7.08 | 8.05 | 5.68 | 6.68 | 7.88 | 8.76 | 11.52 | 11.47 | 11.57 | 7.45 |
| Proportion looking time head female-positive^b^ | 0.04 | 0.04 | 0.04 | 0.04 | 0.03 | 0.04 | 0.06 | 0.06 | 0.08 | 0.08 | 0.08 | 0.09 |
| Dwell time head female-positive (sec)^b^ | 11.26 | 10.54 | 9.02 | 10.28 | 7.80 | 8.12 | 14.66 | 17.29 | 19.59 | 21.74 | 19.60 | 21.83 |
| Proportion looking time head female-negative^b^ | 0.00 | 0.00 | 0.00 | 0.00 | 0.00 | 0.01 | 0.01 | 0.04 | 0.02 | 0.03 | 0.01 | 0.02 |
| Dwell time head female-negative (sec)^b^ | 1.03 | 1.24 | 0.69 | 1.09 | 0.95 | 1.49 | 3.49 | 10.43 | 4.21 | 7.15 | 3.39 | 5.63 |
| Self-focus (single item) | 5.50 | 2.09 | 3.60 | 1.96 | 3.50 | 1.57 | 5.38 | 2.31 | 3.29 | 1.59 | 2.71 | 1.76 |
| External focus (single item) | 5.75 | 2.63 | 7.75 | 1.33 | 6.30 | 1.95 | 5.52 | 2.23 | 6.95 | 1.72 | 6.14 | 2.03 |

***Note.*** The table displays mean values (*M*) and standard deviations (*SD*) for the primary and secondary state outcome variables at pretest, at the intervention, and at posttest for each group. The primary outcomes are underlined. Abbreviations: SUD: subjective units of distress; STAI state: State-Trait Anxiety Inventory, state version; PANAS: Brief Measure of Positive and Negative Affect Schedule; BSQ: Body Sensations Questionnaire; sec: seconds. ^a^ One missing value in the nonsocial focus group, *n*=19, and one in the social focus group, *n*=20. ^b^ One missing value in the nonsocial focus group, *n*=19.

### Supplementary Table 2 │ Comprehensive results for time effects in the state outcome variables between pretest, intervention, and posttest and respective time x group interaction effects for the social vs. nonsocial focus group.

|  | **Pretest to intervention** | | | | | | | **Intervention to posttest** | | | | | | | | **Pretest to posttest** | | | | | |  |
| --- | --- | --- | --- | --- | --- | --- | --- | --- | --- | --- | --- | --- | --- | --- | --- | --- | --- | --- | --- | --- | --- | --- |
|  | **Time effect** | | | **Time x group effect** | | | | **Time effect** | | | | **Time x group effect** | | | | **Time effect** | | | **Time x group effect** | | |  |
|  | *F* | *p* | *η*_p_^2^ | | *F* | *p* | *η*_p_^2^ | | *F* | *p* | *η*_p_^2^ | | *F* | *p* | *η*_p_^2^ | *F* | *p* | *η*_p_^2^ | *F* | *p* | *η*_p_^2^ | |
| SUD | 76.88 | **<.001** | .663 | | 0.28 | .599 | .007 | | 28.71 | **<.001** | .424 | | 2.33 | .135 | .056 | 74.36 | **<.001** | .656 | 0.55 | .462 | .014 | |
| STAI state^a^ | 72.54 | **<.001** | .650 | | 0.11 | .743 | .003 | | 23.82 | **<.001** | .379 | | 2.59 | .116 | .062 | 83.80 | **<.001** | .682 | 1.46 | .234 | .036 | |
| PANAS Positive | 1.39 | .246 | .034 | | 1.91 | .175 | .047 | | 4.10 | **.050** | .095 | | 1.75 | .194 | .043 | 7.73 | **.008** | .165 | 5.57 | **.023** | .125 | |
| PANAS Negative | 32.03 | **<.001** | .451 | | 0.30 | .586 | .008 | | 11.92 | **.001** | .234 | | 3.36 | ***.074*** | .079 | 41.81 | **<.001** | .517 | 0.36 | .553 | .009 | |
| BSQ | 41.90 | **<.001** | .518 | | 4.15 | **.049** | .096 | | 33.65 | **<.001** | .463 | | 0.44 | .512 | .011 | 55.21 | **<.001** | .586 | 3.39 | ***.073*** | .080 | |
| HR^b^ | 36.80 | **<.001** | .499 | | 1.10 | .300 | .029 | | 68.07 | **<.001** | .648 | | 0.28 | .602 | .007 | 74.34 | **<.001** | .668 | 1.41 | .243 | .037 | |
| SLC^b^ | 0.01 | .911 | .000 | | 2.53 | .120 | .064 | | 7.58 | **.009** | .170 | | 0.39 | .539 | .010 | 8.00 | **.008** | .178 | 0.21 | .650 | .006 | |
| Self-rated effect | 29.90 | **<.001** | .434 | | 0.02 | .895 | .000 | | 12.35 | **.001** | .241 | | 0.86 | .361 | .021 | 44.50 | **<.001** | .533 | 0.24 | .627 | .006 | |
| Self-rated appearance^c^ | 35.91 | **<.001** | .479 | | 0.44 | .512 | .011 | | 8.57 | **.006** | .180 | | 0.03 | .858 | .001 | 55.21 | **<.001** | .586 | 0.20 | .654 | .005 | |
| Prop. heads^d,e^ | 1.18 | .285 | .030 | | 14.60 | **<.001** | .278 | | 0.95 | .335 | .024 | | 0.12 | .732 | .003 | 0.27 | .608 | .007 | 21.30 | **<.001** | .359 | |
| Dwt. heads^d^ | 0.03 | .858 | .001 | | 12.97 | **.001** | .254 | | 0.17 | .685 | .004 | | 0.00 | .990 | .000 | 0.02 | .886 | .001 | 20.33 | **<.001** | .349 | |
| Prop. whole bodies^d,f^ | 1.07 | .307 | .027 | | 9.82 | **.003** | .205 | | 1.09 | .302 | .028 | | 0.00 | .975 | .000 | 0.16 | .691 | .004 | 12.95 | **.001** | .254 | |
| Dwt. whole bodies^d,g^ | 0.10 | .758 | .003 | | 4.29 | **.045** | .101 | | 0.00 | .974 | .000 | | 0.59 | .448 | .015 | 0.12 | .728 | .003 | 10.21 | **.003** | .212 | |
| Prop. nonsocial stimuli^d,h^ | 1.62 | .211 | .041 | | 9.74 | **.003** | .204 | | 1.12 | .297 | .029 | | 0.00 | .947 | .000 | 0.35 | .557 | .009 | 11.53 | **.002** | .233 | |
| Dwt. nonsocial stimuli^d,i^ | 9.75 | **.003** | .204 | | 12.37 | **.001** | .246 | | 2.61 | .115 | .064 | | 2.29 | .139 | .057 | 2.41 | .129 | .060 | 4.40 | **.043** | .104 | |
| Prop male/positive^d,j^ | 0.01 | .934 | .000 | | 10.95 | **.002** | .224 | | 0.30 | .590 | .008 | | 0.01 | .944 | .000 | 0.10 | .752 | .003 | 10.40 | **.003** | .215 | |
| Dwt. male/positive^d,k^ | 0.28 | .601 | .007 | | 11.81 | **.001** | .237 | | 0.19 | .668 | .005 | | 0.05 | .829 | .001 | 0.53 | .470 | .014 | 9.80 | **.003** | .205 | |
| Prop. male/negative^d,l^ | 0.02 | .901 | .000 | | 9.61 | **.004** | .202 | | 0.82 | .372 | .021 | | 0.57 | .457 | .015 | 0.23 | .633 | .006 | 14.15 | **.001** | .271 | |
| Dwt. male/negative^d,m^ | 0.17 | .680 | .005 | | 7.88 | **.008** | .172 | | 0.45 | .509 | .012 | | 0.51 | .479 | .013 | 0.82 | .371 | .021 | 11.94 | **.001** | .239 | |
| Prop female/positive^d,n^ | 1.06 | .311 | .027 | | 5.36 | **.026** | .124 | | 0.07 | .801 | .002 | | 0.05 | .834 | .001 | 0.84 | .366 | .022 | 7.69 | **.009** | .168 | |
| Dwt. female/positive^d,o^ | 0.56 | .459 | .014 | | 3.98 | ***.053*** | .095 | | 0.17 | .679 | .005 | | 0.19 | .670 | .005 | 0.27 | .609 | .007 | 8.58 | **.006** | .184 | |
| Prop. female/negative^d,p^ | 0.13 | .726 | .003 | | 0.47 | .495 | .012 | | 0.37 | .544 | .010 | | 2.03 | .162 | .051 | 0.01 | .930 | .000 | 0.00 | .956 | .000 | |
| Dwt. female/negative^d,q^ | 0.03 | .863 | .001 | | 0.23 | .637 | .006 | | 0.39 | .536 | .010 | | 1.46 | .235 | .037 | 0.01 | .936 | .000 | 0.00 | .993 | .000 | |
| Self-focus | 53.32 | **<.001** | .578 | | 0.13 | .723 | .003 | | 2.23 | .144 | .054 | | 1.10 | .301 | .027 | 47.12 | **<.001** | .547 | 0.96 | .333 | .024 | |
| External focus | 24.32 | **<.001** | .384 | | 0.68 | .416 | .017 | | 11.99 | **.001** | .235 | | 0.96 | .332 | .024 | 3.55 | ***.067*** | .083 | 0.01 | .912 | .000 | |

Note. The table displays F-values, p-values, and η_p_^2^ as effect size measure for time effects as well as for time x group interaction effects for the state outcome variables, all within repeated measures ANOVAs. Nonsocial focus group, n=20; social focus group, n=21. Means and standard deviations for both groups and all time points are reported in Supplementary Table 1. Significant p-values (≤.050) are written in bold letters, by trend significant p-values (≤.100) in bold and italic letters. Abbreviations: SUD: subjective units of distress; STAI state: State-Trait Anxiety Inventory, state version; PANAS: Brief Measure of Positive and Negative Affect Schedule; BSQ: Body Sensations Questionnaire; HR: heart rate; SCL: skin conductance level; Prop.: proportion of looking time; Dwt.: dwell time. ^a^ Within the 2x2 ANOVA examining changes in STAI state from pretest to intervention and differences between groups, a significant main effect of group was found, F=5.01, p=.031.^b^ One missing value in the nonsocial focus group, n=19, and one in the social focus group, n=20. ^c^ Within the 2x2 ANOVA examining changes in self-rated appearance from pretest to intervention and differences between groups, a significant main effect of group was found, F=4.36, p=.043. Within the 2x2 ANOVA examining changes in self-rated appearance from pretest to posttest and differences between groups, a significant main effect of group was found, F=4.59, p=.039. ^d^ One missing value in the nonsocial stimuli group, n=19. ^e^ Within the 2x2 ANOVA examining changes in the proportion of looking time on the audience members’ heads from intervention to posttest and differences between groups, a significant main effect of group was found, F=5.10, p=.030. ^f^ Within the 2x2 ANOVA examining changes in proportion of looking time on the audience members’ whole bodies from intervention to posttest and differences between groups, a significant main effect of group was found, F=7.34, p=.010. ^g^ Within the 2x2 ANOVA examining changes in dwell time on the audience members’ whole bodies from intervention to posttest and differences between groups, a significant main effect of group was found, F=4.12, p=.050. ^h^ Within the 2x2 ANOVA examining changes in the proportion of looking time on nonsocial stimuli from intervention to posttest and differences between groups, a significant main effect of group was found, F=6.58, p=.014. ^i^ Within the 2x2 ANOVA examining changes in the dwell time on nonsocial stimuli from pretest to intervention and differences between groups, a significant main effect of group was found, F=5.32, p=.027. Within the 2x2 ANOVA examining changes in the dwell time on nonsocial stimuli from intervention to posttest and differences between groups, a significant main effect of group was found, F=10.91, p=.002. ^j^ Within the 2x2 ANOVA examining changes in the proportion of looking time on the positive male’s head from the intervention to posttest and differences between groups, a significant main effect of group was found, F=4.75, p=.036. ^k^ Within the 2x2 ANOVA examining changes in dwell time on the positive male’s head from the intervention to posttest and differences between groups, a significant main effect of group was found, F=4.45, p=.042. ^l^ Within the 2x2 ANOVA examining changes in the proportion of looking time on the negative male’s head from the intervention to posttest and differences between groups, a significant main effect of group was found, F=5.96, p=.019. ^m^ Within the 2x2 ANOVA examining changes in dwell time on the negative male’s head from the intervention to posttest and differences between groups, a significant main effect of group was found, F=4.09, p=.050. ^n^ Within the 2x2 ANOVA examining changes in the proportion of looking time on the positive females head from the intervention to posttest and differences between groups, a significant main effect of group was found, F=5.12, p=.029. ^o^ Within the 2x2 ANOVA examining changes in the dwell time on the positive females head from the intervention to posttest and differences between groups, a significant main effect of group was found, F=4.63, p=.038. ^p^ Within the 2x2 ANOVA examining changes in the proportion of looking time on the negative females head from the intervention to posttest and differences between groups, a significant main effect of group was found, F=4.41, p=.042. ^q^ Within the 2x2 ANOVA examining changes in dwell time on the negative females head from the intervention to posttest and differences between groups, a significant main effect of group was found, F=4.31, p=.045.

### Supplementary Table 3 │ Group differences at pretest, intervention and posttest.

|  | **Group differences** | | | | | |
| --- | --- | --- | --- | --- | --- | --- |
|  | **Pretest** | | **Intervention** | | **Posttest** | |
| **Variables** | *t* | *p* | *t* | *p* | *t* | *p* |
| SUD | –1.19 | .243 | –1.67 | .103 | –1.20 | .238 |
| STAI state | –2.05 | **.047** | –2.09 | **.043** | –0.94 | .351 |
| PANAS Positive | 1.66 | .104 | 0.80 | .426 | –0.05 | .962 |
| PANAS Negative | –0.80 | .430 | –2.13 | **.040** | –0.55 | .589 |
| BSQ | –1.73 | .092 | –0.67 | .507 | –0.44 | .662 |
| HR^a^ | 0.19 | .853 | –0.47 | .640 | –0.60 | .551 |
| SLC^a^ | 0.08 | .935^c^ | 0.95 | .347 | 0.60 | .554 |
| Self-rated effect | 1.58 | .123 | 2.22 | **.032** | 1.30 | .202 |
| Self-rated appearance | 2.05 | **.045^c^** | 1.64 | .108 | 1.65 | .108 |
| Prop. heads^b^ | 0.61 | .544 | –2.19 | **.035** | –2.18 | **.036** |
| Prop. nonsocial stimuli^b^ | 0.66 | .948 | 2.32 | **.026** | 2.66 | **.011** |
| Prop male-positive^b^ | 2.35 | **.028^c^** | –1.95 | .062^c^ | –2.06 | .051^c^ |
| Prop male-negative^b^ | 1.09 | .281 | –1.76 | .086 | 2.89 | **.010^c^** |
| Prop female-positive^b^ | –0.83 | .411 | –2.26 | **.032^c^** | –2.27 | **.031^c^** |
| Prop female-negative^b^ | –1.06 | .296 | –2.28 | **.033^c^** | –1.88 | .072^c^ |
| Self-focus | 0.17 | .864 | 0.57 | .574 | 1.50 | .141 |
| External focus | 0.30 | .768 | 1.66 | .106 | 0.25 | .802 |
| BFNE | –1.89 | .066 | – | – | –2.26 | **.030** |
| PRCS | –2.80 | **.008** | – | – | –1.24 | .222 |

Note. The table displays t-values and p-values from independent sample t-tests on group differences between the nonsocial focus group (n=20) and the social focus group (n=21) in the state and trait outcome variables at the pretest and posttest speech task, and in state variables also at the interventional speech task. State outcome variables: SUD: subjective units of distress; STAI state: State-Trait Anxiety Inventory, state version; PANAS: Brief Measure of Positive and Negative Affect Schedule; BSQ: Body Sensations Questionnaire; Self rated effect in others; Self-rated appearance; Self-focus; External focus (the last four assessed via single items, score 0–10, each); Prop.: proportion of looking time on different ROIs assessed via eye tracking. Trait outcome variables (measures of general Social Anxiety): BFNE: Brief Fear of Negative Evaluation Scale-Revised; PRCS: Personal Report of Confidence as a Speaker. Significant p-values (≤.050) are written in bold letters. ^a^ One missing value in the nonsocial focus group, n=19, and one in the social focus group, n=20. ^b^ One missing value in the nonsocial focus group, n=19. ^c^ Welch correction due to inhomogeneity of variances according to the Levine test.

### Supplementary Table 4 │ Valence ratings for positive and negative emotional expressions of female and male audience members.

|  | **Total sample (*n*=41)** | |
| --- | --- | --- |
| Variables | *M* | *SD* |
| **Positive emotional expressions** |  |  |
| ***Friendly*** |  |  |
| Male-positive | 8.75 | 1.43 |
| Male-negative | 5.20 | 1.87 |
| Female-positive | 6.58 | 2.24 |
| Female-negative | 2.73 | 2.29 |
| ***Attentive*** |  |  |
| Male-positive | 9.13 | 1.22 |
| Male-negative | 7.25 | 1.94 |
| Female-positive | 7.85 | 1.93 |
| Female-negative | 5.70 | 2.27 |
| ***Positive total*** |  |  |
| Male-positive | 8.94 | 1.19 |
| Male-negative | 6.23 | 1.41 |
| Female-positive | 7.21 | 1.69 |
| Female-negative | 4.21 | 1.82 |
| **Negative emotional expressions** |  |  |
| ***Pejorative*** |  |  |
| Male-positive | 0.68 | 1.90 |
| Male-negative | 2.60 | 2.23 |
| Female-positive | 1.88 | 2.38 |
| Female-negative | 6.23 | 2.45 |
| ***Annoyed*** |  |  |
| Male-positive | 0.70 | 1.88 |
| Male-negative | 2.50 | 2.31 |
| Female-positive | 1.98 | 2.75 |
| Female-negative | 6.75 | 2.76 |
| ***Negative total*** |  |  |
| Male-positive | 0.69 | 1.87 |
| Male-negative | 2.55 | 2.14 |
| Female-positive | 1.93 | 2.50 |
| Female-negative | 6.49 | 2.48 |

***Note.*** Means (*M*) and standard deviations (*SD*) for the participants’ ratings (range 0–10) of the intensity of different emotional expressions of the two female and two male virtual audience members sitting in the first row of the virtual lecture room (Figure 1B), constructed to show either positive valent or negative valent emotional expressions.
